# Seasonal variability in non-consumptive mortality of Arctic zooplankton

**DOI:** 10.1093/plankt/fbab042

**Published:** 2021-06-04

**Authors:** Malin Daase, Janne E Søreide

**Affiliations:** Department of Arctic and Marine Biology, Faculty of Biosciences, Fisheries and Economics, UiT The Arctic University of Norway, PB 6050 Langnes, 9037 Tromsø, Norway; Department of Arctic Biology, The University Centre in Svalbard, PB 156, 9171 Longyearbyen, Norway

**Keywords:** non-predatory mortality, marine copepods, Arctic, polar night, carcasses

## Abstract

Recent observations from high-latitude marine ecosystems indicate that non-consumptive mortality may be particularly high in Arctic zooplankton during the polar night. Here we have estimated the contribution of dead organisms to the mesozooplankton community in the high Arctic (Svalbard 78–81^o^N) during the polar night (January), in spring (May) and in late summer (end of August). To identify *in situ* dead organisms, we used Neutral Red Stain. The dead zooplankton fraction consisted mainly of copepods, while the contribution of dead non-copepods was low in all seasons. The absolute abundance of dead copepods varied little between seasons; however, the relative contribution of dead copepods was highest in January with 11–35% of the copepods classified as dead, in contrast to 2–12% in spring and summer. Furthermore, there were species-specific differences: copepods of the genus *Calanus* contributed more to the dead fraction of the copepod community during the polar night compared to spring and summer, leading to a higher “dead” biomass in winter. We conclude that non-consumptive winter mortality is considerable in calanoid copepods in the Arctic and an important but so far neglected component of the passive carbon flux, providing carbon in larger portions for higher trophic level consumers during the low-productive winter.

## INTRODUCTION

Not all zooplankton are alive in the natural environment, but the contribution of dead individuals to zooplankton populations is normally not accounted for. Samples taken on zooplankton surveys are usually preserved right after collection, while the identification of the non-viable part of the community requires staining prior to fixation or the immediate inspection of freshly caught samples.

Mortality rates of natural plankton populations are notoriously difficult to estimate. They derive indirectly from recruitment rates and changes in abundance, which are subjected to immigration and emigration and thus are often biased. A good understanding of mortality is however important to study population dynamics (Ohman, 2012). Mortality rates do not distinguish between mortality due to predation, which usually does not leave a carcass behind, and non-consumptive mortality, i.e. mortality for other reasons than getting eaten. Hirst and Kiørboe (Hirst and Kiørboe, 2002) estimated that among epipelagic marine copepods 25–40% of mortality may be non-predatory and comparable estimates have been made since (e.g. Elliott and Tang, 2011b; Di Capua and Mazzocchi, 2017; Maud *et al.*, 2018). While the reasons for non-consumptive mortality are often difficult to determine, it is relatively straightforward to observe—at least until the carcass has been consumed or decomposes. Neglecting the contribution of dead organisms can lead to erroneous estimations of abundances, biomass and productivity and may bias our understanding of population dynamics and energy fluxes in aquatic ecosystems (Elliott and Tang, 2011a; Frangoulis *et al.*, 2011; Jónasdóttir *et al.*, 2019). Zooplankton carcasses may also play an important role in aquatic food webs and the carbon cycle. They provide a substrate for bacteria, may promote nutrient retention within the water column (Tang *et al.*, 2006b; Tang *et al.*, 2009; Dubovskaya *et al.*, 2015) and contribute to the formation of macro-aggregates and thus to the detrital pool of aquatic systems (Simon *et al.*, 2002; Isinibilir *et al.*, 2011), and they may serve as nutritious food for benthic organisms (e.g. Søreide *et al.*, 2013; Jónasdóttir *et al.*, 2015). Together with algae aggregates, marine snow, fecal material produced by zooplankton and fish (Turner, 2015; Saba *et al.*, 2021), zooplankton carcasses are likely an important but rarely quantified source to the passive carbon flux (Sampei *et al.*, 2009b; Sampei *et al.*, 2012).

While the occurrence of zooplankton carcasses has been reported from many marine [reviewed in Daase *et al*. (Daase *et al*., 2014)] and freshwater habitats [reviewed in Tang *et al*. (Tang *et al*., 2014)], the reason for non-consumptive mortality is often unknown. In marine habitats, the occurrence of zooplankton carcasses has been associated with, for example, senescence (Sampei *et al.*, 2012), low-oxygen layers (Elliott *et al.*, 2013), thermoclines (Terazaki and Wada, 1988), upwelling (Weikert, 1977), salinity gradients (Isinibilir *et al.*, 2011) as well as river run off and glacial meltwater (Weslawski and Legezynska, 1998; Eiane and Daase, 2002). The majority of observations of non-consumptive mortality are from lower latitudes (reviewed in Daase *et al.*, 2014) with only three studies reporting on copepod carcasses from the Arctic (Sampei *et al.*, 2009b; Sampei *et al.*, 2012; Daase *et al.*, 2014).

High latitudes are characterized by an extreme seasonality in incoming solar radiation, leading to prolonged periods (up to 6 months) when the sun does not rise (polar night, October–March) or does not set (April–September). Daase *et al*. (Daase *et al*., 2014) found high abundance of dead *Calanus* spp. during the polar night (early January) in the European Arctic, with 9–94% of the population observed dead. While death after reproduction and partial consumption could be ruled out, the causes for this mortality could not be determined in that study. Sampei *et al*. (Sampei *et al*., 2009b) estimated that copepod carcasses contribute 36% to the particle carbon flux in the Canadian Arctic, with the contribution of copepod carcasses to the particle flux being higher in winter–early spring (16–91%) compared to the rest of the year (1–30%), largely due to death after reproduction by *Calanus hyperboreus* (Sampei *et al.*, 2012). These observations suggest that the abundance of dead zooplankton may be particularly high during the polar night and in winter at high latitudes. Giesecke *et al*. (Giesecke *et al*., 2017) also observed increased zooplankton mortality in winter in a mid-latitude Chilean estuary. In contrast, Elliott and Tang (Elliott and Tang, 2011b) found higher numbers of carcasses of *Acartia tonsa* during summer compared to winter in the estuaries of Chesapeake Bay, while Maud *et al*. (Maud *et al*., 2018) observed similar non-consumptive mortality rates of *Calanus helgolandicus* in the North Sea in summer and winter, but a decrease during autumn. Di Capua and Mazzocchi (2017) found highest abundance of copepod carcasses from spring to autumn in the Mediterranean, while other studies from lower latitudes (e.g. Terazaki and Wada, 1988; Yamaguchi and Ikeda, 2001) did not report seasonal differences in the occurrence of carcasses.

The extreme light climate at high latitudes leads to resource limitation for a large part of the year, and many Arctic zooplankton species rely heavily on the short but intense spring bloom for reproduction, growth and development. This includes primarily herbivorous species, such as copepods of the genus *Calanus*, who dominate the Arctic zooplankton community in terms of biomass, as well as more omnivorous copepods such as *Oithona similis*, *Pseudocalanus* spp. and *Microcalanus* spp., who dominate the zooplankton community in terms of numbers (Daase and Eiane, 2007; Kosobokova *et al.*, 2011). To deal with the seasonal resource limitation, Arctic zooplankton species have evolved special adaptations, such as prolonged life cycles, energy storages in the form of lipids and overwintering phases (Ejsmond *et al.*, 2018; Berge *et al.*, 2020).

Despite such adaptations, winter mortality may be considerable in some species. For example, the abundance of *Calanus* copepods usually decreases drastically (up to 90%) from late autumn to spring (e.g. Madsen *et al.*, 2001; Arnkværn *et al.*, 2005; Leu *et al.*, 2011; Daase *et al.*, 2013).

Since we first estimated the amount of *Calanus* carcasses in Svalbard waters in January 2012 (Daase *et al.*, 2014), we repeatedly observed copepod carcasses during consecutive polar night field campaigns in the same study area, indicating that this is a reoccurring feature of the Arctic pelagic ecosystem during the polar night. Recent observations indicate that biological activity during the polar night is much higher than that previously assumed (Berge *et al.*, 2015). How this activity is sustained in the absence of primary production remains unclear. Zooplankton carcasses may account for a relatively high but so far unaccounted proportion of biomass in the water column during the polar night and may therefore be an important carbon source for microbial and/or benthic activity during the polar night. Furthermore, adding just a low mortality rate leads to a 12–25% increase in the estimated respired carbon by diapausing copepods in northern temperate seas (Jónasdóttir *et al.*, 2015, 2019).

As there is a lack of observations of non-consumptive mortality in the Arctic from other seasons, it is unclear if the occurrence of zooplankton carcasses is a particular phenomenon of the polar night or if it is a common feature year-round. The aim of this study was therefore to estimate the abundance and proportion of dead zooplankton in different seasons and discuss reasons for potential seasonal differences. Specifically, we wanted to clarify if the occurrence of dead zooplankton is higher during the polar night than during the light season and if there are species-specific differences in the occurrence and contribution of carcasses between seasons. Copepods of the genus *Calanus* spp. are key species in Arctic pelagic ecosystems, dominating the mesozooplankton community in terms of biomass (Conover, 1988; Kosobokova and Hirche, 2009) and serving as important food sources for zooplankton, fish, seabirds and marine mammals (Falk-Petersen *et al.*, 1990). As our previous observations showed high non-consumptive mortality in *Calanus* spp. during the polar night, another aim of our study was to improve our understanding of what may cause this high non-consumptive winter mortality in *Calanus* spp., specifically, if it indeed would differ between seasons. We therefore also estimate seasonal changes in the stage-specific non-consumptive mortality of *Calanus* spp. and analyze seasonal changes in lipid reserves to elucidate if insufficient energy reserves may contribute to high winter mortality.

## METHODS

### Study area

**Fig. 1:**
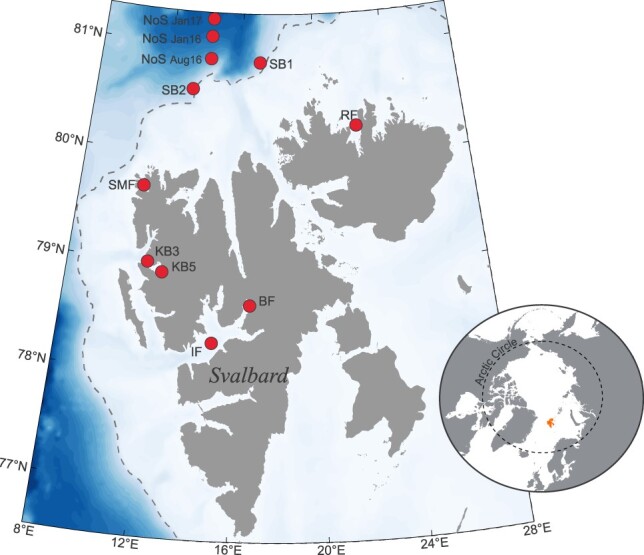
Map of study area and position of stations sampled in January, May and August 2016 and January 2017 (see Supplementary Table SI for details). IF Isfjorden, BF Billefjorden, KB3/KB5 Kongsfjorden, SMF Smeerenburgfjorden, RF Rijpfjorden. NoS North of Svalbard, SB Shelf break. Dotted line marks 500 m bathymetry line.

The abundance of dead and live mesozooplankton was estimated in January, May and August 2016 and in January 2017, thus roughly following one annual cycle. In the study area, the polar night lasts from mid-November to mid-February, placing the January sampling in the middle of the polar night. The May sampling occurred prior or during the pelagic spring bloom, which can occur between April–July in the study area. The August sampling took place toward the end of the midnight sun period (end of August), placing it seasonally at the end of summer/early autumn. Zooplankton samples were collected in fjords along the western and northern coast of the Svalbard archipelago and off-shelf north of Svalbard (78–81^o^N, Fig. 1, Supplementary Table SI) on board R/V Helmer Hanssen. Altogether, 10 different locations were sampled: in January 2016, samples were collected in Billefjorden (BF), Isfjorden (IF), in the middle (KB3) and the innermost part (KB5) of Kongsfjorden (KF), in Smeerenburgfjorden (SMF), Rijpfjorden (RF), north of Svalbard beyond the shelf break (NoS) and at two locations located along the shelf break (SB1 and SB2) (Fig. 1). In May and August 2016, sampling was repeated in BF, IF, KB3 and SMF, with additional sampling in August 2016 in KB5, RF and NoS (although not at the exact same position as NoS in January 2016). In January 2017, sampling was repeated in IF, KB3, SMF, RF and NoS (Fig. 1, Supplementary Table SI).

Isfjorden (IF) and Kongsfjorden (KF) are open fjords located on the western coast of Spitsbergen being influenced by advection of both Atlantic water from the West Spitsbergen Current and Arctic water from the Coastal Current (Cottier *et al.*, 2005; Skogseth *et al.*, 2020). Billefjorden is a sill fjord located in the inner part of the Isfjorden system with a basin (maximum depth 190 m) dominated by locally formed cold water (<−0.5°C year-round) providing a refuge for Arctic zooplankton species (Arnkværn *et al.*, 2005). Smeerenburgfjorden is located at the northwestern corner of Spitsbergen and is influenced by Atlantic water as well as glacial input. Rijpfjorden is a north-facing fjord dominated by cold Arctic water masses, but inflow of Atlantic water may occur (Wallace *et al.*, 2010). The West Spitsbergen Current (WSC) transports Atlantic water along the west coast of Svalbard and splits into two branches at the northwestern corner of Svalbard. One of these branches, the Svalbard branch, turns eastwards and enters the Arctic Ocean following the continental slope (Rudels *et al.*, 1999). The offshore stations (NoS, SB1 and SB2) were located within that path. All stations were ice-free during our sampling campaigns in January, May and August.

### Hydrography

Prior to zooplankton sampling, temperature and salinity were measured from surface to bottom by a ship-board conductivity, temperature and depth profiler (SBE911, Sea-Bird Electronics, Bellevue, WA, USA) at all stations.

### Zooplankton sampling

**Fig. 2:**
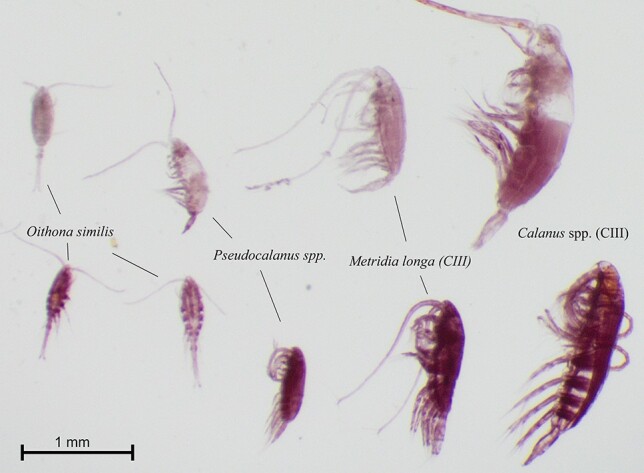
Images of four copepod species stained with Neutral Red Stain. Copepods with coloration as depicted in upper row were identified as dead; the lower row shows coloration in copepods that were alive during the staining process. Photo: courtesy of Slawomir Kwasniewski.

Zooplankton was sampled by vertical hauls (towing speed 0.5 m s^−1^) from close to the seafloor to the surface using a multiple opening/closing net (Multinet, Hydrobios, Kiel, mouth opening 0.25 m^2^, mesh size 180 μm). Due to time constrains, replicate sampling was not possible. Up to five depth strata were sampled at each location (Supplementary Table SI). Samples were dyed with Neutral Red Stain following procedures described in Elliott and Tang (Elliott and Tang, 2009), before being preserved in a 4% formaldehyde-in-seawater solution. Samples were stored in the dark and analyzed within four months after sampling. Dead and live organisms were distinguished by their color (Fig. 2). The interpretation of the red coloration is not always unambiguous and may vary with taxonomic group, zooplankton density and preservation method (Elliott and Tang, 2009; Di Capua and Mazzocchi, 2017). All samples in our study were analyzed by the same person, so while there may be a potential to misinterpret the coloration, any bias should be constant between samples. If in doubt, an individual was regarded as “alive”. Furthermore, low temperatures and the use of Neutral Red Stain on formalin-fixed samples may reduce the staining efficiency and lead to an underestimation of the number of dead individuals (Elliott and Tang, 2009). Thus we may potentially have underestimated the number of carcasses, but the seasonal patterns should not be affected. Organisms may also perish during sampling, e.g. if nets are towed too fast or flushed with too high water pressure, and if samples are exposed to fresh water or stored too long in room temperature before being processed, and thus bias the estimate of *in situ* dead. We took precautions to treat the samples gently and processed them directly after sampling. Furthermore, sampling was conducted in a similar manner and by the same person during all four cruises; thus, if death was caused during sampling, we would expect similar contributions of dead zooplankton in all season.

For species determination and enumeration, large (total length > 5 mm) organisms were removed from the entire sample and identified and counted. The remaining part of the sample was examined by sub-sampling with aliquots obtained with 5 mL automatic pipette, with the pipette tip cut at 5 mm diameter to allow free collection of mesozooplankton. The number of sub-samples analyzed was chosen so that at least 150 individuals of *Calanus* spp. and 300 other copepods were counted. Samples with low abundance were examined in their entirety.

The prosome length of all counted individuals of *Calanus* spp. was measured from the tip of the cephalosome to the distal lateral end of the last thoracic segment. To distinguish between the morphologically similar *Calanus glacialis* and *C. finmarchicus*, we used size classes derived for each developmental stage (copepodites CI-CVI) from prosome length frequency analyses for the study region (Daase *et al.*, 2018). Molecular studies have shown a high, but regionally variable, overlap in prosome length between the two species (Gabrielsen *et al.*, 2012; Choquet *et al.*, 2017). This causes potential bias toward an underestimation of *C. glacialis* and a comparative overestimation of *C. finmarchicus* in our study area. Since the carcasses of *Calanus* spp. were in different stages of decomposition and thus making it even harder to avoid misidentification based on size alone, we did not determine *Calanus* spp. carcasses within the size class of *C. finmarchicus* and *C. glacialis* to species but reported them combined as *Calanus* spp. carcasses.

Zooplankton abundance was estimated by multiplying mouth-opening area of the net by vertical hauling distance assuming 100% filtration efficiency. Abundance estimates were converted to biomass using taxon- and stage-specific dry weights (DWs) and weight–length relationships provided in Hop *et al*. (Hop *et al*., 2019b).

### Data analysis

Since abundance and biomass varied considerably between stations and seasons, the relative contribution of carcasses (percent dead) per station was calculated for the different zooplankton taxa. We grouped copepods into either *Calanus* spp. or “Other Copepods”.

Generalized linear models (GLMs) were applied to identify the main predictors of the depth integrated abundance (ind m^−2^) and biomass (mg DW mg^−3^) of carcasses and percent dead of *Calanus* spp. and Other Copepods, as well as abundance of carcasses and percent dead of *Pseudocalanus* spp., *Oithona similis* and *Microcalanus* spp. Sampling month (SM; January 2016, May 2016, August 2016 and January 2017) and water mass (WM) were included as explanatory variables in the GLM. WM was determined following water mass definitions by Cottier et al. (Cottier et al., 2005) as Arctic Water (ArW, *T* < 1°C, *S* < 34.65), Surface Water (SW, *T* > 1°C, *S* > 34), Intermediate water (IW, *T* > 1°C, *S* 34.0–34.65), Transformed Atlantic Water (TAW, *T* = 1–3°C, *S* > 34.65) or Atlantic Water (AW, *T* > 3°C, *S* > 34.65) based on average temperature and salinity over the sample interval. Furthermore, we used GLMs to explore which environmental parameter best predict the depth resolved carcass abundance (ind m^−3^) and percent dead of *Calanus* spp. and Other Copepods. In addition to SM and WM, sampling layer (SL) was included as explanatory variable (5-level factor, 0–20 m, 20–50 m, 50–100 m, 100–200 m/bottom, 200 m-bottom).

All statistical analyses were done in R (version 3.6.1) (R Core Team, 2020) using R Studio (version 1.2.5001). Data distribution was investigated using the fitdistrplus package in R (Delignette-Muller and Dutang, 2015). Abundance and biomass of carcasses followed a gamma distribution, while the proportional data followed a beta distribution. Consequently, the GLMs for abundance and biomass were run with Gamma family and a logit link using the glm function in R. For the proportional data, we use the betareg package in R (Cribari-Neto and Zeileis, 2010). The most parsimonious model was selected using Akaike’s Information Criterion (Burnham and Anderson, 2002) corrected for small sample size (AICc, R-package AICcmodavg; Mazerolle, 2020). The model with the smallest AICc, and/or the most parsimonious, i.e. other models with ΔAICc <2 and lower k, was chosen as the best model.

To describe where in the water column the live and dead zooplankton were centered, we calculated the weighted mean depth (*Z*_m_) and the standard deviation (*Z*_s_) of the frequency distribution throughout the water column following equations described in Daase *et al*. (Daase *et al*., 2016). To compare *Z*_m_ between stations with highly variable bottom depth (60–1600 m), we calculated the relative weighted mean depth (*RZ*_m_) as the ratio of weighted mean depth to the depth of the deepest sample at each station. Normality and homogeneity of the data were confirmed by the Shapiro Wilk and Bartlett test, respectively. We performed a two-way ANOVA to test if the *RZ*_m_ of *Calanus* spp. and of Other Copepods was different between the live and dead fraction and between sampling month.

To compare the *Calanus* spp. copepodite stage composition of the live and dead fraction, we calculated a stage index (SI) for both the live and dead part of the *Calanus* spp. population (*C. finmarchicus* and *C. glacialis*) at each station as follows:

$$ \mathrm{SI}=\frac{\sum_{j=1}^n{a}_j\ast{b}_j}{\sum_{j=1}^n{a}_j} $$

where *a_j_* is the abundance of copepodite stage *j*, and *b* the copepodite stage as a numeric value with CI = 1, CII = 2, CIII = 3, CIV = 4, CV = 5, females = 6, males = 7. The stage index varies between 1 and 7, with low values indicating a dominance of young copepodite stages, and high values pointing to a dominance of older copepodite stages and/or adults.

Normality and homogeneity of the data were confirmed by the Shapiro Wilk and Bartlett test, respectively. To test if the stage index differed between the life and dead part of the population in May 2016, one-way ANOVA was performed. For January 2016, August 2016 and January 2017 visual inspection of the data indicated difference in stage composition between stations along the west coast of Svalbard (BF, IF, KF, SMF) and stations sampled in the north (NoS, SB, RF). To test if the stage index differed between the live and dead part of the population, and between western and northern locations, we performed a two-way ANOVA.

### Estimation of lipid content

To estimate seasonal changes of lipid content of *Calanus* spp. additional Multinet samples were taken in BF, IF, KB3, SMF, RF and NoS in January 2016; in BF, KB3, RF and SMF in August 2016 and in IF in May 2016. Digital images (lateral view) of all live *Calanus* spp. specimens in sub-samples containing at least 100 *Calanus* spp. were taken following procedures described in Daase *et al*. (Daase *et al*., 2014) using a Leica stereomicroscope with a camera (Leica DFC420). The copepodite stage of each individual was determined while taking the pictures. Lipid sac area and prosome length of specimens were measured from digital images using ImageJ (Rasband, 1997–2009). Lipid and wax ester content of individual *Calanus* specimens were calculated from lipid sac area according to Vogedes *et al*. (Vogedes *et al*., 2010). In live *Calanus* spp., the coloration of the antennules can be used to distinguish between *C. glacialis* and *C. finmarchicus* (Nielsen *et al.*, 2014; Choquet *et al.*, 2018) and we used this criteria to assign lipid content to species. Shapiro Wilk test and Bartlett test suggested non-normality and heterogeneity in the lipid data. We therefore use the non-parametric Kruskal–Wallis test followed by the *post hoc* test according to Nemenyi for pairwise multiple comparisons of the ranked data to test for differences in lipid contesnt of copepodite stages CIV and CV and adult females between seasons.

## RESULTS

### Physical environment

Relatively warm and saline Atlantic or transformed Atlantic (1.5–4°C) water prevailed in January in both years (Supplementary Fig. S1), except for BF, RF and SMF where colder Arctic waters (<0°C) dominated in both years. In May, the water column was well mixed and colder than in January, with transformed Atlantic water (0.5–1.5°C) dominating from bottom to surface, except in Billefjorden where cold locally formed Arctic waters still prevailed. In August, the water column was strongly stratified with warm (4–6°C) and less saline surface waters (33–34.2) with transformed Atlantic waters or intermediate waters found below the surface layers in most location, except for BF and the deeper layers in RF where Arctic waters prevailed.

### Zooplankton composition

The live and dead fraction of the zooplankton community was numerically dominated by copepods (Table I). In January 2016 and 2017, non-copepod taxa (mainly chaetognaths in the fjords, and ostracods offshore) accounted for only 1–7% of the live zooplankton abundance (Supplementary Fig. S2). In spring and summer, the contribution of non-copepod taxa to the live fraction was higher and much more variable between stations (6–93% in May, 11–56% in August), due to the presence of meroplankton taxa in spring (particularly cirripedia nauplii) and juvenile pteropods *Limacina helicina* in August (Supplementary Table SII). Among non-copepod taxa, only chaetognaths and euphausiids were observed dead. In May and August <1% of non-copepod taxa were classified as dead (Table I). In January 2016, >93% of non-copepods were alive, except for KB5 where 32% of non-copepods, mainly chaetognaths, were found dead. In January 2017, 1.5–12% of non-copepods (also mainly chaetognaths) were classified dead (Table I). Since the abundance of non-copepod taxa was generally low and non-copepods contributed little to the dead fraction (with exceptions), we restricted the following analyses to copepods only.

**Table I:** Abundance (ind m^−2^), biomass (mg DW m^−2^) of live and dead zooplankton at each station, number of dead individuals and contribution (%) of *Calanus* spp., Other Copepods and non-copepod taxa to the dead fraction in terms of abundance and biomass

	Abundance (ind m^−2)^			Composition (%) of dead fraction			Biomass (mg DW m^−2^)			Composition (%) of dead fraction			Number of dead individuals per station		
Station	Total live	Total dead	% dead	*Calanus* spp.	Other Copepods	Non-copepods	Total live	Total dead	% dead	*Calanus* spp.	Other Copepods	Non-copepods	*Calanus* spp.	Other Copepods	Non-copepods
January 2016															
BF	319 928	40 516	11.2	20.6	79.4	<0.01	31 872	1692	5.0	85.3	14.5	0.2	2083	8045	1
IF	78 959	12 424	13.6	25.0	75.0	0.0	9922	745	7.0	89.8	10.2	0.0	777	2329	0
KB3	37 038	14 370	28.0	15.5	84.4	0.1	5500	624	10.2	71.2	25.0	3.8	556	3032	5
KB5	21 812	8505	28.1	70.8	27.4	1.7	1671	2159	56.4	90.5	2.8	6.7	1506	583	37
SMF	37 460	16 744	30.9	10.2	89.8	0.0	2537	566	18.2	66.2	33.2	0.6	428	3758	1
RF	86 714	21 798	20.1	59.2	40.4	0.4	13 396	4675	25.9	95.2	1.8	3.0	3228	2199	22
NoS	61 532	20 100	24.6	30.0	69.6	0.4	6058	2290	27.4	91.4	5.3	3.3	1508	3499	18
SB1	83 321	17 470	17.3	50.1	49.9	<0.05	7908	3643	31.5	97.4	2.1	0.5	2191	2178	2
SB2	28 720	10 560	26.9	53.3	45.0	1.7	4987	2578	34.1	83.8	8.5	7.7	1407	1189	44
Mean	**83 943**	**18 054**	**22.3**	**37**	**62**	**1**	**9317**	**2108**	**24.0**	**86**	**11**	**3**			
SD	91 852	9470	7.0	22	22	1	9193	1406	16.2	11	11	3			
May 2016															
BF	114 197	4416	3.7	0.0	100.0	0.0	4361	41	0.9	0.0	100.0	0.0	0	1104	0
IF	94 980	2040	2.1	0.6	98.2	0.0	3732	20	0.5	33.2	66.2	0.0	3	507	0
KB3	212 257	9477	4.3	11.0	83.7	3.0	9414	647	6.4	92.3	6.8	0.7	261	2037	71
SMF	456 615	880	0.2	16.5	78.0	5.5	9474	128	1.3	60.4	5.8	33.8	36	172	12
Mean	**219 512**	**4203**	**2.6**	**7.0**	**90.0**	**2**	**6745**	**209**	**2.3**	**46.5**	**44.7**	**8.6**			
SD	166 202	3812	1.8	8.1	10.8	3	3127	296	2.8	39.3	46.4	16.8			
August 2016															
BF	363 760	16 730	4.4	2.9	96.9	0.2	31 733	206	0.6	47.5	35.0	17.5	120	4053	10
IF	237 256	21 040	8.1	16.2	79.7	3.9	25 197	1504	5.6	43.7	7.2	49.1	851	4204	205
KB3	407 655	19 519	4.6	14.7	84.9	0.4	26 803	1083	3.9	75.4	18.3	6.3	719	4142	19
KB5	1 613 445	67 095	4.0	2.9	96.9	0.2	33 938	815	2.3	59.7	27.1	13.2	131	4334	8
SMF	530 544	13 856	2.5	1.8	98.2	0.0	34 152	144	0.4	44.9	55.1	0.0	62	3402	0
RF	454 439	21 256	4.5	14.6	84.9	0.5	42 019	978	2.3	78.4	12.2	9.4	786	4513	24
NoS	471 596	23 004	4.7	28.1	71.8	0.0	25 967	847	3.2	92.4	7.6	0.0	1618	4133	0
Mean	**582 671**	**26 071**	**4.7**	**11.6**	**87.6**	**1**	**31 401**	**797**	**2.6**	**63.1**	**23.2**	**13.6**			
SD	464 015	18 350	1.7	9.7	10.1	1	6000	482	1.8	19.2	17.4	16.9			
January 2017															
IF	51 528	7721	13.0	34.8	65.1	0.2	6038	657	14.3	89.7	8.7	1.6	671	1 256	3
KB3	18 229	9356	33.9	22.1	76.3	1.6	2081	737	33.9	63.7	17.4	18.8	518	1784	37
SMF	22 281	4113	15.5	18.8	80.9	0.3	2933	296	11.5	79.0	11.7	9.3	193	832	3
RF	41 856	10 487	20.0	10.4	88.0	1.6	3087	483	16.8	47.0	14.7	38.3	273	3 591	42
NoS	32 317	12 040	27.1	36.2	60.9	2.9	6079	1 183	21.5	57.7	12.5	29.9	1 089	1833	87
Mean	**33 242**	**8743**	21.9	**24**	**74**	**1**	**4044**	**671**	19.6	**67**	**13**	**20**			
SD	13 738	3032	8.6	11	11	1	1879	333	8.8	17	3	15			

Mean of all stations (in bold) and standard deviation (SD) given for each month

### Copepod community

**Fig. 3:**
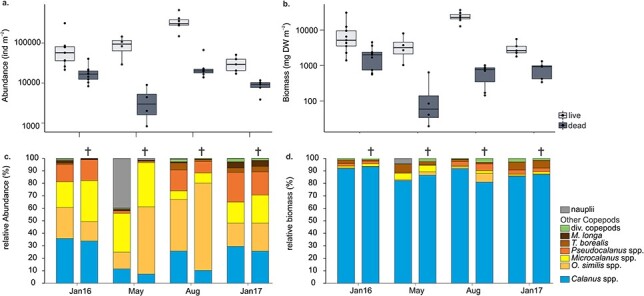
Differences in (**a**) abundance (ind m^−2^) and (**b**) biomass (mg DW m^−2^) of live and dead copepods, and species composition of the live and dead (bars with cross on top) fraction of the copepod community in January, May and August 2016 and January 2017 based on (**c**) abundance and (**d**) biomass; mean of all stations for each month (see Table I for standard deviations). div. copepods—all other copepod species identified. Note log scale on *y*-axis in (a) and (b). Dots in (a) and (b) show data points.

Abundance of live and dead copepods was highest in August. Highest dead copepod biomass was observed in January 2016 (Fig. 3a and b), and lowest dead copepod abundance and biomass in May. In terms of abundance, dead copepods contributed between 11 and 31% to the total copepod community in January 2016 and 13–35% in January 2017, but only 2–6% in May and 5–12% in August. In terms of biomass, dead copepods contributed 5–59, 1–8, 1–5 and 11–34% to the total copepod biomass in January, May, August 2016 and January 2017, respectively.

The live and dead fraction of the copepod community at all stations and seasons was dominated by *Calanus* spp., *Microcalanus* spp., *Pseudocalanus* spp. and *O. similis* (Fig. 3c and d). Together, these species accounted for 89–99% of the copepod community. *Triconia borealis* and *Metridia longa* were commonly observed but generally in low abundance (<5%), both in the live and dead fraction. Other copepod species contributed little to the live or dead fraction (<2.5 and <1.5%, respectively; Fig. 3c and d). The relative species composition of the dead and live fraction of copepods was similar in January 2016 and 2017 except for a slightly higher contribution of *Microcalanus* spp. to the dead fraction compared to the live. In May, the live fraction was dominated by copepod nauplii, while the dead fraction comprised mainly of *Microcalanus* spp. and *O. similis* (Fig. 3c). In August, the dead fraction was comprised of a larger proportion of *O. similis* compared to the live fraction. In terms of biomass, *Calanus* spp. dominated both the live and dead fraction of the copepod community year-round (80–93%, Fig. 3d).

Carcass abundance, biomass and percent dead of *Calanus* spp., Other Copepods, as well as carcass abundance and percent dead of *Pseudocalanus* spp., *Microcalanus* spp. and *O. similis* were best explained by sampling month while the inclusion of water mass did not improve model performance (Supplementary Table SII).

**Fig. 4:**
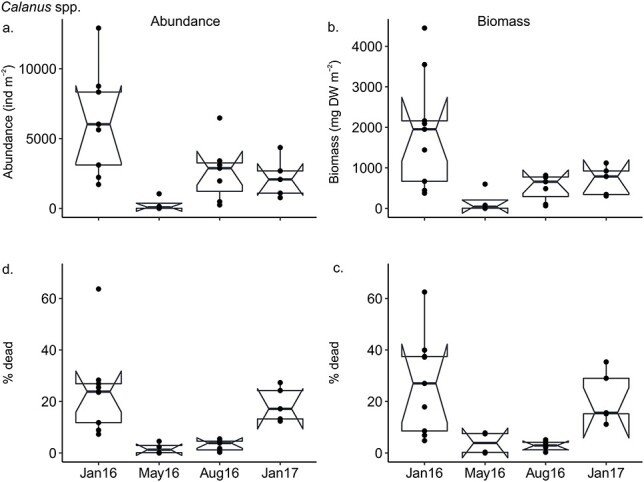
Differences in (**a**) abundance (**b**) biomass (**c**) percent dead in terms of abundance and (**d**) percent dead in terms of biomass of dead *Calanus* between January, May and August 2016 and January 2017. Note differences in scale of *y*-axis in b. Horizontal line shows median, the bottom and top of the box show the 25 and 75 percentiles, respectively. Whiskers extend 1.5 times the interquartile range of the sample. Values outside this range are marked by circles. The boxes are drawn with widths proportional to the square-roots of the number of observations in the groups. Notches display the variability of the median between samples. The width of a notch is computed so that box plots whose notches do not overlap have different medians (Chambers *et al.*, 1983).

The abundance and biomass of dead *Calanus* spp. significantly differed among sampling month (Table II) and were highest in January 2016 compared to the other three sampling months (Fig. 4a and b). The percent dead *Calanus* spp. was significantly higher in both January 2016 and 2017 compared to May and August in terms of abundance and compared to August in terms of biomass (Table II, Fig. 4c and d).

**Table II:** Parameter estimates for the generalized linear model (GLM) that best predicts the carcass abundance and biomass as well as the percent dead (based on abundance and biomass) of *Calanus* spp., Other Copepods, *Pseudocalanus* spp., *Oithona similis* and *Microcalanus* spp

	Abundance					Biomass	
	*Calanus* spp.	Other Copepods	*Pseudocalanus* spp.	*O. similis*	*Microcalanus* spp.	*Calanus* spp.	Other Copepods
Intercept	6080.37^***^	11 916.05^***^	3005.81^***^	2792.88^***^	5920.25^***^	1903.93^***^	136.53^***^
	[3406.67, 10 852.49]	[7480.79, 18 980.94]	[1757.84, 5139.78]	[1521.94, 5125.14]	[3569.78, 9818.35]	[1074.77, 3372.76]	[94.21, 197.86]
May 2016	0.05^***^	0.32^*^	0.01^***^	0.79	0.25^**^	0.09^***^	0.19^***^
	[0.02, 0.14]	[0.14, 0.73]	[0.01, 0.04]	[0.27, 2.37]	[0.10, 0.61]	[0.03, 0.25]	[0.10, 0.38]
August 2016	0.44	1.95	0.80	6.49^***^	0.35^*^	0.28^**^	0.90
	[0.18, 1.05]	[0.97, 3.94]	[0.36, 1.80]	[2.59, 16.25]	[0.16, 0.76]	[0.12, 0.65]	[0.51, 1.58]
January 2017	0.36	0.53	0.53	0.69	0.32^*^	0.37	0.76
	[0.14, 0.95]	[0.24, 1.16]	[0.22, 1.30]	[0.25, 1.90]	[0.14, 0.76]	[0.14, 0.95]	[0.41, 1.41]
*N*	25	25	25	25	25	25	25
AIC	450.07	512.43	413.48	476.52	453.43	390.44	272.96
pseudo *R*^2^	0.44	0.52	0.67	0.59	0.38	0.41	0.46
Percent Dead							
Intercept	−1.19^***^	−1.27^***^	−0.84^***^	−1.93^***^	−0.52^**^	−1.11^***^	−1.40^***^
	[−1.57, −0.80]	[−1.56, −0.98]	[−1.25, −0.44]	[−2.26, −1.60]	[−0.88, −0.15]	[−1.57, −0.65]	[−1.73, −1.07]
May 2016	−2.32^***^	−0.91^**^	−2.00^***^	0.31	−1.50^***^	−1.89^***^	−0.99^**^
	[−3.37, −1.27]	[−1.54, −0.28]	[−3.01, −0.99]	[−0.24, 0.87]	[−2.33, −0.67]	[−2.96, −0.81]	[−1.72, −0.26]
August 2016	−1.83^***^	−0.95^***^	−1.68^***^	0.01	−1.54^***^	−1.73^***^	−1.07^***^
	[−2.62, −1.03]	[−1.47, −0.43]	[−2.47, −0.90]	[−0.49, 0.50]	[−2.22, −0.86]	[−2.60, −0.87]	[−1.68, −0.47]
January 2017	−0.18	0.11	−0.10	0.73^**^	−0.39	−0.11	−0.07
	[−0.84, 0.47]	[−0.36, 0.58]	[−0.78, 0.59]	[0.25, 1.22]	[−1.03, 0.24]	[−0.87, 0.66]	[−0.62, 0.48]
phi	14.60^***^	28.66^***^	10.92^***^	31.90^***^	12.59^***^	9.34^***^	23.07^***^
	[6.01, 23.20]	[12.81, 44.52]	[4.71, 17.13]	[14.26, 49.54]	[5.68, 19.51]	[3.86, 14.82]	[10.21, 35.94]
nobs	25	25	25	25	25	25	25
pseudo *R*^2^	0.67	0.54	0.72	0.29	0.52	0.57	0.45
df.null	23.00	23.00	23.00	23.00	23.00	23.00	23.00
logLik	38.64	33.74	28.27	35.24	22.66	33.21	34.02
AIC	−67.29	−57.49	−46.54	−60.47	−35.32	−56.42	−58.04
df.residual	20.00	20.00	20.00	20.00	20.00	20.00	20.00

For model selection, see Supplementary Table SIII. Predictor variable is sampling month (January 2016, May 2016, August 2016 and January 2017) with January 2016 set as reference (Intercept). Confidence intervals are given in squared brackets. GLM of abundance and biomass was run with a gamma distribution, percent dead (abundance and biomass) with beta regression. ^***^P < 0.001; ^**^P < 0.01; ^*^P < 0.05

**Fig. 5:**
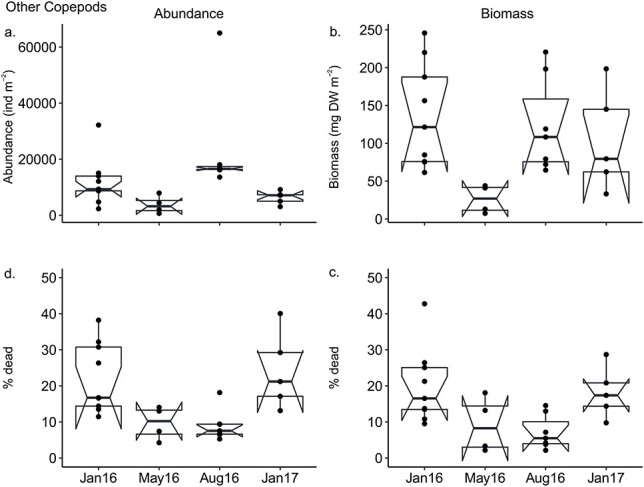
Differences in (**a**) abundance (**b**) biomass (**c**) percent dead in terms of abundance and (**d**) percent dead in terms of biomass of dead Other Copepods between January, May and August 2016 and January 2017. Note differences in scale of *y*-axis for b. Horizontal line, whiskers, circles and notches as in Fig. 4.

The total abundance and biomass of dead Other Copepods significantly differed between the four sampling months, with high dead abundance in August and low dead biomass in May 2016 (Fig. 5a and b; Table II). The percent dead of Other Copepods, both in terms of abundance and biomass, was significantly higher in January 2016 and 2017 compared to August (Fig. 5c and d).

**Fig. 6:**
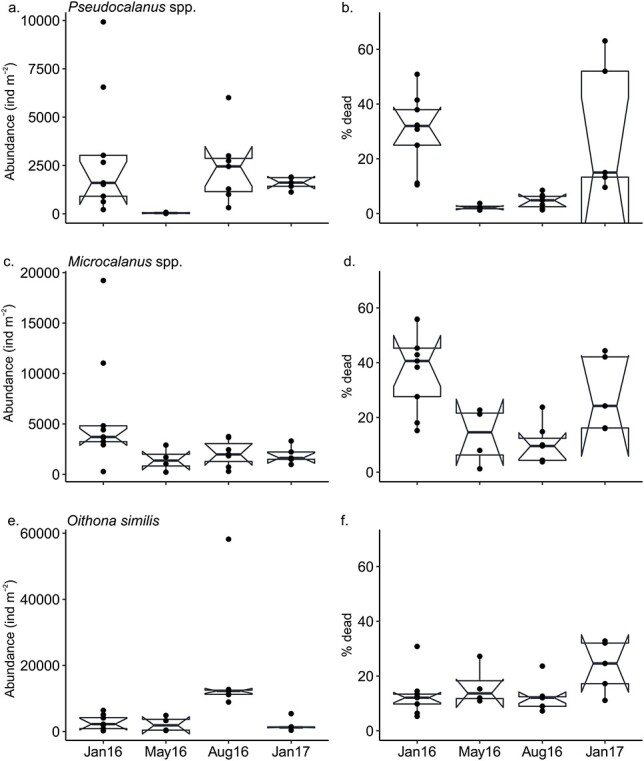
Difference in abundance (left panel) and percent dead (right panel) of (a, b) *Pseudocalanus* spp., (c, d) *Microcalanus* spp. and (e, f) *Oithona similis* between January, May and August 2016 and January 2017. Horizontal line, whiskers, circles and notches as in Fig. 4.

The abundance of dead *Pseudocalanus* spp. was significantly lower in May, and the percent dead was significantly lower in May and August compared to January (Fig. 6a, Table II). Carcasses of *Microcalanus* spp. were more abundant in January 2016, and the percent dead of this species was significantly lower in May and August compared to January (Fig. 6b). The abundance of *O. similis* carcasses was higher in August compared to January, and the percent dead of *O. similis* was highest in January 2017 (Fig. 6c).

### *Calanus* stage composition

The abundance of *C. hyperboreus* was low (<2% of *Calanus* abundance) and the majority of *Calanus* species were within the size range of *C. glacialis* and *C. finmarchicus,* hereafter termed *Calanus* spp.

**Fig. 7:**
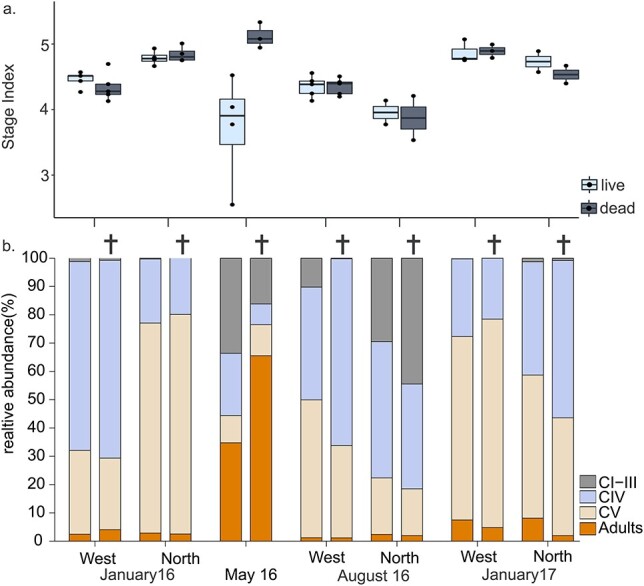
(**a**) Differences in *Calanus* spp. stage index between live and dead fraction of the population and (**b**) stage composition of live and dead (bars with cross on top) fraction of the *Calanus* spp. community at stations along the western coast and north of Svalbard in January, May (no northern stations) and August 2016, and January 2017. Based on mean abundance (ind m^−2^) between stations. Dots in (a) show data points.

There were no differences in the stage composition between dead and live fraction of the *Calanus* spp. population. Overwintering stages CIV and CV of *Calanus* spp. made up the majority of both the dead and live fraction in January 2016 and 2017 (Fig. 7), while the contribution of younger copepodite stages (CI–III) was low (<2%) in January of both years. Length frequency distributions of the live fraction showed that CVs in January were mainly *C. finmarchicus,* while CIVs and adults were identified as *C. glacialis* (see also Daase *et al.*, 2018). Differences in the stage index (SI) between the dead and live fraction were not significant. However, there were significant differences in SI between western and northern stations in January 2016 (two-way ANOVA, *F* = 0.314, *P* = 0.583 and *F* = 31.848, *P* > 0.001) but not in 2017 (two-way ANOVA, *F* = 0.357, *P* = 0.569 and *F* = 5.088, *P* > 0.001), with a dominance of CIVs (*C. glacialis*) in the west and a dominance of CVs (*C. finmarchicus)* in the north in 2016.

In May, the *Calanus* spp. population comprised of both younger stages (CI–III) and adults (Fig. 7). Adults contributed to a larger extend to the dead fraction (>60%) and differences in the SI of the dead and live fraction were significant (ANOVA, *F* = 7.619, *P* = 0.0398). In August, overwintering stages CIV and CV dominated, especially in the western stations, while CI–IIIs made up a larger proportion of the live fraction of the population in the northern stations (~30%). The dead *Calanus* spp. fraction comprised of both CIV (66%) and CVs (33%) in the west, while a high proportion of dead CI–III was observed in the north (~44%, Fig. 7). Differences in the *Calanus* spp. stage index of the dead and live fraction were not significant in August, but there were significant differences between western and northern stations (two-way ANOVA, *F* = 0.045, *P* = 0.837 and *F* = 12.725, *P* = 0.004) (Fig. 7).

### Vertical distribution

The vertical distribution of live and dead copepods was highly variable between stations in each season (Supplementary Fig. S3). In January, copepods were distributed throughout the water column, with higher concentration in the upper 50 m in some locations (KB3, RF and north of Svalbard) and concentrated at depth in others (BF, SMF). In May, copepods were concentrated at the surface except for BF where the majority was found in the deepest layer. In August, higher abundance was observed at the surface and in the deepest layer, and lower abundance at intermediate depth.

Sampling month was also the best explanatory variable of depth resolved abundance of dead *Calanus* spp. as well as percent dead of *Calanus* spp. and Other Copepods. Only for the depth resolved abundance of dead Other Copepods did model performance improve by including water mass as an explanatory variable (Supplementary Tables SIV and SV). This was largely driven by high abundance in surface water in August in the western fjords, which was also observed in the live fraction of the population. Sampling layer did not improve model performance. There were no significant differences in the *RZ*_m_ of *Calanus* spp. and Other Copepods between the live and dead fraction and between sampling month (*P* > 0.05). Only the *RZ*_m_ of live *Calanus* spp. differed between seasons (*F* = 5.341, *P* = 0.007), with *Calanus* spp. being located higher up in the water column in May compared to August and January (Supplementary Fig. S4).

### Lipid content of live Calanus

**Fig. 8:**
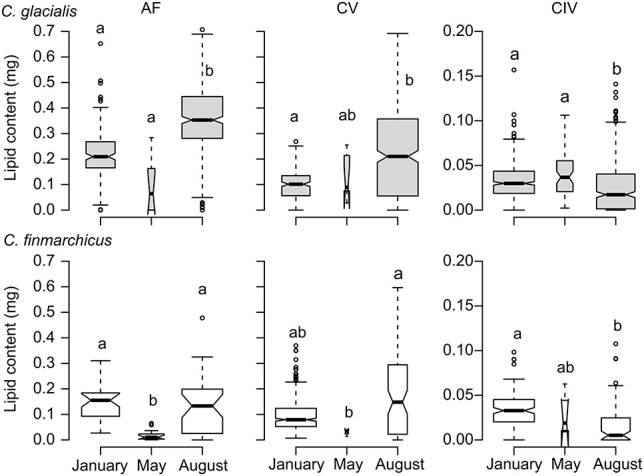
Boxplot of lipid content (mg) of adult females (AF) and copepodite stages CIV and CV of live *C. glacialis* (gray) and *C. finmarchicus* (white) in January, May and August 2016. Different letters indicate significant differences between month (Kruskal–Wallis and *post hoc* tests according to Nemenyi, *P* < 0.05). Note differences in scale of y-axis for CIV. Horizontal line, whiskers, circles and notches as in Fig. 4.

Mean lipid content of *C. glacialis* CVs and adult females (AF) was highest in August, with lowest mean lipid content observed in May for AF and in January for CVs (Fig. 8, Supplementary Table SVI). There was no difference in mean lipid content of AF and CVs of *C. finmarchicus* between August and January, and lowest lipid content was observed in May. Lipid content of CIVs of both species was lowest in August, while there were no differences between January and May. In May 2016, lipid content was measured on a much smaller number of individuals than during the other three sampling campaigns (Supplementary Table SVI), so these results have to be taken with caution.

## DISCUSSION

The aim of our study was to estimate the abundance and biomass of dead zooplankton in the Arctic, to clarify if the occurrence of dead zooplankton is higher during the polar night than during the light season and if there are species-specific differences. We found a higher percentage of dead copepods during the polar night compared to May and August. Particularly, the calanoid copepods *Calanus* spp., *Microcalanus* spp. and *Pseudocalanus* spp. suffered higher non-consumptive mortality in January than in May and August. The total abundance of copepod carcasses was not higher during the polar night compared to spring and summer, but due to the high proportion of relatively large dead *Calanus* specimens in January, the dead copepod biomass was overall higher during the polar night than during spring and summer when the abundance of dead *Calanus* spp. was low. The observed percentage of dead copepods and particularly that of *Calanus* spp. in January is within the range of the mean percentage of dead marine zooplankton (12–60%) estimated by (Tang *et al.*, 2014), while our spring and summer values are below the ranges observed during summer surveys elsewhere [e.g. see Table I in Daase *et al*. (Daase *et al*., 2014)]. The high contribution of *Calanus* spp. carcasses in January is in agreement with previous observations in the study area in January 2012 (Daase *et al.*, 2014), and with observations from the Canadian Arctic reporting high abundance of carcasses of *Calanus* spp. during the polar night and in late winter (Sampei *et al.*, 2009b).

The main causes for non-consumptive mortality in zooplankton in this study likely differ between seasons and seem to be more related to copepods’ life history strategies and energy demand rather than to direct environmental stressors. Copepod carcasses were found throughout the water column and were not accumulated at depth, water mass was generally a poor predictor of carcass abundance and there was also no difference in the depth distribution between the live and dead fraction of the copepod population. This indicates that water mass properties and hydrographic forcing such as oxygen minimum layers, pycnoclines or osmotic stress in freshwater layers are unlikely causes of mortality. However, we cannot exclude that carcasses were re-suspended from the sea floor due to strong vertical mixing caused by extreme weather events or the local current system. Our study area included a variety of fjord and oceanic locations with different bathymetry and currents regimes, as well as stretching over a large geographical region experiencing different weather conditions. The vertical distribution of carcasses is likely affected differently in these different locations. Studies with higher temporal resolution that also assess the abundance of carcasses on the sea floor are needed to resolve the likelihood and frequency of re-suspension events.

The occurrence of zooplankton carcasses in high-latitude glacial fjords has been associated with glacial melt water causing increased mortality due to osmotic shock (Hartley and Fisher, 1936; Weslawski and Legezynska, 1998). While none of our sampling sites were close to a glacier front, all fjords included in this study are subjected to glacial runoff during summer, and we can’t exclude that carcasses could have been advected from freshwater zones close to the glaciers. However, if mortality was caused by glacial runoff, we would expect to find high numbers of carcasses in late summer when runoff is highest, which was not the case. Maud *et al*. (Maud *et al*., 2018) related high wind speeds to increased non-consumptive mortality in *C. helgolandicus* in the North Sea suggesting that strong turbulence in the water column caused by storms may promote mortality. Similar observations have been made in freshwater systems [reviewed in Tang *et al*. (Tang *et al*., 2014)]. Winter storms are common occurrences in January in our study area, and since copepods were abundant in surface layers at this time, extreme episodic weather events may very well play a role in enhancing non-consumptive morality during winter, but this needs to be addressed in more detail in future studies.

### Non-consumptive mortality in Calanus spp

A decrease in population size of Arctic and boreal *Calanus* species of >90% from autumn to spring has repeatedly been reported (e.g. Madsen *et al.*, 2001; Arnkværn *et al.*, 2005; Leu *et al.*, 2011; Daase *et al.*, 2013). As the life span of *C. finmarchicus* and *C. glacialis* in the Arctic is 1 and 1–2 years, respectively (Falk-Petersen *et al.*, 2009), and they perish after reproduction, a decrease in population size during the main reproductive season is to be expected, and the low *Calanus* spp. abundance in May in our study supports this. But a decrease in population size is often already observed prior to the spring bloom and before the onset of the main reproductive period (Madsen *et al.*, 2001, Arnkværn *et al.*, 2005, Leu *et al.*, 2011; Daase *et al.*, 2013) indicating that winter mortality is considerable in *Calanus* spp. Predation risk should theoretically be lower during the polar night given the absence of migrating predators, such as baleen whales and planktivorous seabirds, and the reduced visibility in the absence of sunlight. However, recent studies have shown that visual predators are successfully catching plankton also during the polar night (Kraft *et al.*, 2013; Berge *et al.*, 2015). Thus predation likely contributes to the decline in population size during winter, but the presence of carcasses during winter observed in our study indicates that non-predatory mortality is also an important factor. While parasites in copepods may cause high mortality (Kimmerer and Mckinnon, 1990; Walkusz and Rolbiecki, 2007), the carcasses and live copepods observed in our study did not show any obvious parasitic ailments.

Arctic and boreal *Calanus* species have evolved different strategies to cope with the seasonal resource limitation in high latitudes, including prolonged life cycles, energy storages in the form of lipids and an overwintering phase in a state of diapause at depth (Hirche, 1996; Ejsmond *et al.*, 2018). One re-occurring observation during polar night field campaigns, which we also observed in this study, is that both *C. finmarchicus* and *C. glacialis* are distributed all over the water column already in early January and do not reside at greater depth in a state of diapause (Daase *et al.*, 2014; Berge *et al.*, 2015; Basedow *et al.*, 2018; Daase *et al.*, 2018). This suggests that they emerge from overwintering depth and become active long before the spring bloom. *Calanus* spp. is regarded as primarily herbivorous, but their feeding strategies are flexible and they can switch to an omnivorous diet when phytoplankton concentration is low (e.g. Levinsen *et al.*, 2000; Campbell *et al.*, 2009; Cleary *et al.*, 2017). While a recent model study indicates that an early emergence from overwintering depth does not necessarily decrease fitness as long as a low concentration of some food source is available (Hobbs *et al.*, 2020), it still raises the question if energy reserves are sufficient to sustain activity throughout late winter until the spring bloom commences in early May in the study area (Hegseth *et al.*, 2019).

Based on wax ester content derived from lipid sac area according to Vogedes *et al*. (Vogedes *et al*., 2010), and assuming a wax ester–lipid carbon conversion factor of 0.8 (Kattner and Hagen, 2009), we can estimate the lipid carbon content of *C. finmarchicus* and *C. glacialis* (Supplementary Table SVI). Reported respiration rates for CVs of these two species vary from 0.4–0.8 μg C d^−1^ in winter for *C. finmarchicus* (Jónasdóttir *et al.*, 2015) and *C. glacialis* (Morata and Søreide, 2013), to 5.5 μg C d^−1^ in summer (*C. glacialis*; Morata and Søreide, 2013). At low respiration rates (0.4–0.8 μg C d^−1^), lipid reserves accumulated by CVs in August may last for 104–209 and 214–429 days, for *C. finmarchicus* and *C. glacialis*, respectively. However, lipid content varied considerably between individuals. For those individuals with high lipid content in August, lipids could theoretically last 439–879 days (*C. finmarchicus*) and 608–1217 (*C. glacialis)*, while those with the lowest lipid content may run out of lipid reserves after 8–15 days and 79–157, respectively. Thus the observed non-consumptive mortality may partly be explained by resource depletion by those individuals with low lipid reserves at the start of the overwintering phase. Furthermore, energetic costs significantly increase in January due to ascending, molting, mating and gonad maturation that commence after organisms emerge from diapause (Jónasdóttir, 1999). As soon as metabolic activities increase, the lipid carbon may quickly be respired. With a mean lipid carbon content of 68 μg C in CV *C. glacialis* in January 2016, lipid reserves may only last 12–17 days at respiration rates of 5.5 μg C d^−1^.

Mean and maximum lipid content of CVs and AF of *C. glacialis* was significantly lower in January than in August (Fig. 8), indicating that lipids are utilized during the polar night. However, mean lipid content of CVs and AF of *C. finmarchicus* did not differ significantly between August and January. Maps *et al*. (Maps *et al*., 2014) showed that the metabolic rate of *C. finmarchicus* during diapause was among the lowest measured. This may explain lower lipid lost in *C. finmarchicus* compared to *C. glacialis* and may indicate species-specific differences in non-consumptive mortality. However, length frequency distribution of live and dead CIVs and CVs in January 2017 showed significantly smaller body size of the dead fraction (Supplementary Fig. S5), suggesting higher mortality in the smaller *C. finmarchicus*. But length measurements of *Calanus* spp. carcasses in different states of decomposition are not a good species indicator, and molecular species identification is needed in future studies to resolve if there are species-specific differences in *Calanus* spp. mortality.

If we assumed that individuals with low lipid content suffer higher non-consumptive mortality during autumn, we can expect that these individuals with low lipid content will perish first. Indeed, our data show that the August population of *C. finmarchicus* CV consisted to a higher degree of individuals with low lipid content (CV: 1st quartile 10 μg) compared to January (1st quartile 53 μg in January 2016¸ Fig. 8, Supplementary Table SVI). Thus, in January, the population consisted mainly of individuals with high lipid content, while the ones with low lipid content had vanished, resulting in similar mean lipid content in January and August. Similarly, mean lipid content of CIVs of both species was higher in January compared to August and did not decrease significantly between January and May. However, the *C. glacialis* population in August contained more CIVs with low lipid content (1st quartile 6 μg; Fig. 8, Supplementary Table SVI), which had disappeared by January (1st quartile 19 μg), indicating that individuals with low lipid content were less abundant in the population and likely had perished.

Not unexpected, we observed lowest lipid content in May, reflecting the utilization of lipids to fuel reproduction. Similar observations have been made previously in Arctic *Calanus* populations (e.g. Lee, 1974; Søreide *et al.*, 2010; Wold *et al.*, 2011).

### Calanus spp. stage-specific mortality

Different life stages of *Calanus* spp. are likely to be affected differently by non-consumptive mortality, as for example senescence and death after reproduction will target adults to a higher degree than juveniles (Hatlebakk *et al.*, 2019). We did not observe stage-specific non-consumptive mortality in *Calanus* spp. in January. In both years, the *Calanus* spp. population was dominated by overwintering stages CIV and CV, although the contribution of adults was slightly higher in January 2017 (5%) compared to 2016 (3%). However, overall abundance was much lower in 2017 than in 2016, indicating that significant mortality may have occurred earlier that winter. Water temperatures in January 2017 were overall warmer than in January 2016 (Supplementary Fig. S1). As metabolism and energetic costs as well as molting rates from CV to adults increase with temperature, differences in stage composition and abundance between January 2016 and 2017 may be explained by the differences in water temperatures between the years.

In May, the dead fraction was characterized by a higher proportion of adults compared to the live fraction, indicating death after reproduction as the most likely cause of death. However, the number of *Calanus* spp. carcasses was generally low in May (Table I), so any interpretations have to be taken with caution.

By late August, the *Calanus* spp. population was again dominated by overwintering stages and had descended to overwintering depth. High proportions of young copepodite stages (CI–CIII) within the dead fraction observed in August likely reflect increased mortality in these late recruits as they may struggle to find enough food so late in the season and thus fail to reach the overwintering stage and are lost from the population.

### Non-consumptive mortality of Other Copepods

Among the Other Copepods, we observed species-specific differences in non-consumptive mortality between seasons. Similar to *Calanus* spp., a higher percentage of the dead *Pseudocalanus* spp. and *Microcalanus* spp. was observed in January. *Pseudocalanus* spp. has similar life history strategies to *Calanus* spp., being primarily herbivorous and relying on stored lipids during winter (Lischka and Hagen, 2005, 2007). This indicates an increased susceptible to non-consumptive mortality during winter in primarily herbivorous species and that winter mortality may be connected to resource limitation.

Both *Microcalanus spp.* and *O. similis* are omnivorous species that remain active year-round and reproduce during winter (Ashjian *et al.*, 2003; Darnis and Fortier, 2014). However, *O. similis* usually remains in surface layers where food source may be more abundant in winter than at depth where *Microcalanus* spp. resides (Darnis and Fortier, 2014). This difference in depth distribution may explain why *Microcalanus* spp. suffered increased non-consumptive mortality in winter and spring, and why *O. similis* did not, although slightly high percent dead of *O. similis* was observed in January 2017.

### Importance of copepod carcasses for the marine ecosystem

Copepod carcasses may at times represent a substantial carbon source in the water column compared to what else is available (Tang and Elliott, 2014). Carcasses also provide carbon in larger portions, making it available for larger consumers that cannot utilize particulate carbon. Sampei *et al*. (Sampei *et al*., 2009a) suggested that copepod carcasses present a high-quality food source for pelagic predators, such as omnivorous and carnivorous copepods, particularly in winter when carbon sources in the water column consisted largely of recycled and degraded matter (Forest *et al.*, 2007). Converting our estimated *Calanus* spp. carcasses biomass to carbon using a dry weight to carbon conversion factor of 0.5 (Richter, 1994), the carbon standing stock in the upper 50 m provided by *Calanus* spp. carcasses varied between 7–445 mg C m^−2^ in winter, 0.6–22 mg C m^−2^ in spring and 7–165 mg C m^−2^ in late summer. These values are likely overestimations since we did not correct for decomposition. However, maximum winter values of carbon provided by carcasses are up to a fifth of the POC estimates by Iversen and Seuthe (Iversen and Seuthe, 2010) for the upper 50 m in Kongsfjorden in December (2150 mg C m^−2^). In spring and summer, the contribution of *Calanus* spp. carcasses to the carbon pool on the other hand is negligible (<0.1 and <3.2%, in May and August, respectively) compared to the POC estimates for Kongsfjorden (32 350 mg C m^−2^ in May, and 5300 mg C m^−2^ in September; Iversen and Seuthe, 2010).

Zooplankton carcasses function as microbial hotspots (Tang *et al.*, 2006b; Bickel and Tang, 2010) and may function as microsites for denitrification (Glud *et al.*, 2015; Stief *et al.*, 2018). Not all carcasses will be recycled within the water column. The amount that is retained in the water column or that is sinking to deeper layers and the sea floor depends on decomposition time and sinking velocity. Stepanov and Svetlichny (1981, cited in Isinibilir *et al.*, 2011) described decomposition time as a function of temperature, and based on their equation, the decomposition of copepod carcasses would take 17–31 days within the temperature range encountered in our study area (−1 to 6°C). However, low temperatures slow decomposition considerably (Terazaki and Wada, 1988; Tang *et al.*, 2006a, 2006b). Terazaki and Wada (Terazaki and Wada, 1988) suggested that carcasses of *Calanus cristatus* in the Japan Sea may drift for more than a year due to slow decomposition rates at low ambient temperatures (2°C), and reduced predation pressure and slow decomposition may lead to high abundance of copepod carcasses in the deep sea (Yamaguchi *et al.*, 2002). As sinking speed depends on particle size and density (Dubovskaya *et al.*, 2015), carcasses of smaller, less dense species sink slowly and may therefore largely be decomposed by microbes within the water column (Tang *et al.*, 2009), while larger, denser carcasses will sink faster and contribute to a large degree to the flux of organic matter to the deep (Sampei *et al.*, 2009a; Ivory *et al.*, 2014). *Calanus* spp. carcasses have been observed with lipid remnants (Daase *et al.*, 2014), which may render them neutrally buoyant, preventing them from sinking out or slowing down sinking. This could explain why we did not observe *Calanus* spp. carcasses accumulated at depth. However, sediment trap studies have demonstrated that *Calanus* spp. carcasses will eventually sink to deeper layers in winter (Sampei *et al.*, 2009b). Given the low water temperatures and the lack of stratification in the water column in January, we can assume that *Calanus* spp. carcasses we observed may sink before they are decomposed. The fatty acid composition of many benthic invertebrates in Svalbard waters show high relative contributions of the *Calanus* spp. specific long chain monounsaturated fatty acids and fatty alcohols 20:1 and 22:1, suggesting benthic invertebrates feed on *Calanus* spp. or on prey that have fed on *Calanus* spp. (Søreide *et al.*, 2013). With a mean abundance of ~4000 ind m^−2^ of *Calanus* spp., carcasses in the water column in January, and assuming a lipid carbon content of ~70 μg per individual, *Calanus* spp. carcasses would account for ~290 mg C per m^−2^ that could potentially sink to the seafloor providing a nutritious food source in an otherwise low productive season.

While our observations indicate high non-consumptive mortality of *Calanus spp.* and small calanoid copepods during the polar night compared to the light season, we can only estimate the amount of non-consumptive mortality as long as we find carcasses. Given the higher visibility during the light season, carcasses may be subjected to higher predation in spring and summer. Decomposition may also be higher during spring and summer due to increased microbial activity, i.e. carcasses are not preserved in the water column as long in spring and summer as during winter. Thus there is a potential for underestimating the occurrence of carcasses in summer.

## CONCLUSION

*Calanus* spp. and copepod species with similar life history strategies as *Calanus* spp. suffered higher non-consumptive mortality in January compared to the more productive light season. Vertical distribution patterns of live *Calanus* spp. show that they do not reside at overwintering depth in January, indicating that diapause is terminated long before the onset of the spring bloom. Changes in population lipid content between August and January indicate that individuals with low lipid content disappear from the population during the polar night. We therefore suggest that insufficient energy stores to sustain activities throughout winter largely contribute to non-consumptive mortality in winter. However, since dead individuals have been observed with lipid sac still intact (Daase *et al.*, 2014), insufficient lipid reserves may not be the only cause of non-consumptive mortality in winter (e.g. Hatlebakk *et al.*, 2019). Better estimates of the energetic costs of overwintering are needed to resolve how long lipid reserves can sustain the organisms. With global warming, water temperatures are increasing (Walczowski *et al.*, 2017), also during winter (Hop *et al.*, 2019a; Skogseth *et al.*, 2020). If insufficient energy reserves contribute significantly to the non-consumptive mortality, we may expect an increase in winter mortality with increasing winter temperatures as this will lead to increased metabolic rates putting further constraints on energy reserves. Our estimates indicate that *Calanus* spp. carcasses contributed substantially to the carbon pool in winter. The role of carcasses for the carbon cycle, i.e. for the lipid pump (Jónasdóttir *et al.*, 2015; Jónasdóttir *et al.*, 2019), needs to be addressed in future studies, as this may be a missing puzzle piece in understanding Arctic marine ecosystems functioning during the polar night.

## Supplementary Material

Supplementary_material_Daase_Soreide_fbab042
