# Supplementary material for: Seasonal variability in non-consumptive mortality of Arctic zooplankton
Source: J Plankton Res. 2021 Jun 4;43(4):565–85. doi: 10.1093/plankt/fbab042 (PMC8315232; doi:10.1093/plankt/fbab042)
Supplement: Supplementary_material_Daase_Soreide_fbab042 [file supplementary_material_daase_soreide_fbab042.docx]

**Seasonal variability in non-consumptive mortality of Arctic zooplankton**

Malin Daase, Janne E. Søreide

**SUPPLEMENTARY MATERIAL**

**Supplementary Table SI:** Overview of stations, sample date and sample depth

| Station | Latitude | Longitude | Bottom depth (m) | Date | Time (UTC) | Sampling depth intervals (m) |  |
| --- | --- | --- | --- | --- | --- | --- | --- |
| January 2016 |  |  |  |  |  |  |  |
| Isfjorden (IF) | 78^o^ 19.2 | 15^o^ 09.7 | 277 | 11.01.2016 | 06:36:22 | 258-200-100-50-20-0 |  |
| Billefjorden (BF) | 78^o^ 39.6 | 16^o^ 43.8 | 192 | 11.01.2016 | 15:42:35 | 180-150-100-50-20-0 |  |
| Kongsfjorden (KB3) | 78^o^ 57.4 | 11^o^ 57.4 | 341 | 20.01.2016 | 10:10:02 | 320-200-100-50-20-0 |  |
| Kongsfjorden (KB5) | 78^o^ 53.8 | 12^o^ 26.5 | 81 | 21.01.2016 | 14:11:03 | 60-50-20-0 |  |
| Smeerenburgfj. (SMF) | 79^o^ 41.8 | 11^o^ 06.1 | 215 | 12.01.2016 | 22:18:28 | 200-150-100-50-20-0 |  |
| Rijpfjorden (RF) | 80^o^ 18.2 | 22^o^ 16.0 | 283 | 14.01.2016 | 06:33:24 | 265-200-100-50-20-0 |  |
| North of Svalbard (NoS) | 80^o^ 58.1 | 14^o^ 57.7 | 1627 | 15.01.2016 | 02:42:51 | 1600-1000-600-200-50-0 |  |
| Shelf break (SB1) | 80^o^ 50.8 | 16^o^ 56.5 | 608 | 15.01.2016 | 08:13:37 | 585-200-100-50-20-0 |  |
| Shelf break (SB2) | 80^o^ 44.7 | 13^o^ 59.3 | 591 | 16.01.2016 | 00:38:56 | 550-200-100-50-20-0 |  |
| May 2016 |  |  |  |  |  |  |  |
| Isfjorden (IF) | 78^o^ 18.9 | 15^o^ 07.8 | 277 | 15.05.2016 | 12:41:26 | 260-200-100-50-20-0 |  |
| Billefjorden (BF) | 78^o^ 39.6 | 16^o^ 43.8 | 192 | 15.05.2016 | 21:06:15 | 180-150-100-50-20-0 |  |
| Kongsfjorden (KB3) | 78^o^ 57.4 | 11^o^ 57.4 | 341 | 11.05.2016 | 10:42:41 | 300-200-100-50-20-0 |  |
| Smeerenburgfj. (SMF) | 79^o^ 42.2 | 11^o^ 05.7 | 215 | 12.05.2016 | 10:05:38 | 165-100-50-20-0 |  |
| August 2016 |  |  |  |  |  |  |  |
| Isfjorden (IF) | 78^o^ 18.9 | 15^o^ 07.8 | 277 | 30.08.2016 | 23:06:25 | 260-200-100-50-20-0 |  |
| Billefjorden (BF) | 78^o^ 39.6 | 16^o^ 43.8 | 192 | 31.08.2016 | 04:05:37 | 180-150-100-50-20-0 |  |
| Kongsfjorden (KB3) | 78^o^ 57.4 | 11^o^ 57.4 | 341 | 22.08.2016 | 09:53:49 | 320-200-100-50-20-0 |  |
| Kongsfjorden (KB5) | 78^o^ 53.8 | 12^o^ 26.5 | 81 | 23.08.2016 | 20:25:32 | 60-50-20-0 |  |
| Smeerenburgfj. (SMF) | 79^o^ 42.2 | 11^o^ 05.7 | 215 | 26.08.2016 | 04:54:00 | 208-150-100-50-20-0 |  |
| Rijpfjorden (RF) | 80^o^18.2 | 22^o^ 16.0 | 283 | 27.08.2016 | 01:14:12 | 268-200-100-50-20-0 |  |
| North of Svalbard (NoS) | 80^o^ 44.7 | 15^o^ 29.1 | 1336 | 28.08.2016 | 22:51:46 | 1200-800-600-200-50-0 |  |
| January 2017 |  |  |  |  |  |  |  |
| Isfjorden (IF) | 78^o^ 18.9 | 15^o^ 07.8 | 278 | 08.01.2017 | 02:45:04 | 260-200-100-50-20- |  |
| Kongsfjorden (KB3) | 78^o^ 57.4 | 11^o^ 57.4 | 341 | 09.01.2017 | 12:57:56 | 320-200-100-50-20-0 |  |
| Smeerenburgfj. (SMF) | 79^o^ 42.2 | 11^o^ 05.7 | 211 | 16.01.2017 | 02:27:44 | 190-100-50-20-0 |  |
| Rijpfjorden (RF) | 80^o^ 18.2 | 22^o^ 16.0 | 277 | 14.01.2017 | 18:01:27 | 250-200-100-50-20-0 |  |
| North of Svalbard (NoS) | 81^o^ 22.3 | 14^o^ 49.7 | 2281 | 13.01.2017 | 00:00:11 | 1600-1200-800-400-200-100-50-20-0 |  |

**Supplementary** **Table SII**: Mean abundance (ind m^-2^) and standard deviation (SD) of life and dead zooplankton species and life stages identified in locations sampled in January 2016 (mean of 9 stations), May 2016 (4 stations), August 2016 (7 stations) and January (6 stations).

|  |  | January 2016 | | May 2016 | | August 2016 | | January 2017 | |
| --- | --- | --- | --- | --- | --- | --- | --- | --- | --- |
| *Species* | **Stage** | **mean** | **SD** | **mean** | **SD** | **mean** | **SD** | **mean** | **SD** |
| LIVE |  |  |  |  |  |  |  |  |  |
| *Acartia longiremis* |  | 239.8 | 511.1 | 148.3 | 96.3 | 3255.7 | 3898.9 | 46.9 | 71.7 |
| *Aetideidae* indet. | CI-CIII | 0.0 | 0.0 | 0.0 | 0.0 | 0.0 | 0.0 | 4.0 | 8.9 |
| *Bradydius similis* |  | 245.4 | 471.7 | 377.5 | 495.0 | 1773.3 | 1548.6 | 47.2 | 79.3 |
| *Calanus finmarchicus* | AM | 3.1 | 7.9 | 149.0 | 227.0 | 0.6 | 1.5 | 40.0 | 0.0 |
| *Calanus finmarchicus* | AF | 112.0 | 67.6 | 2245.0 | 3543.3 | 560.4 | 892.2 | 2412.5 | 52.8 |
| *Calanus finmarchicus* | CV | 9995.0 | 8569.2 | 306.7 | 228.5 | 13632.5 | 7670.7 | 3102.9 | 3067.9 |
| *Calanus finmarchicus* | CIV | 2257.6 | 1303.9 | 75.0 | 54.0 | 18729.9 | 17644.2 | 732.3 | 1624.8 |
| *Calanus finmarchicus* | CIII | 18.7 | 41.2 | 0.0 | 0.0 | 3943.6 | 4290.6 | 1.9 | 5.2 |
| *Calanus finmarchicus* | CII | 0.0 | 0.0 | 71.7 | 116.6 | 1516.9 | 983.2 | 0.0 | 0.0 |
| *Calanus finmarchicus* | CI | 0.0 | 0.0 | 2222.3 | 2652.2 | 912.7 | 659.4 | 121.2 | 0.0 |
| *Calanus glacialis* | AM | 275.1 | 380.3 | 52.0 | 72.5 | 1.7 | 3.1 | 314.0 | 187.8 |
| *Calanus glacialis* | AF | 365.8 | 319.6 | 1164.7 | 1449.7 | 975.1 | 788.1 | 560.0 | 303.4 |
| *Calanus glacialis* | CV | 3451.9 | 5048.4 | 687.8 | 446.3 | 18219.8 | 10240.6 | 1103.7 | 929.4 |
| *Calanus glacialis* | CIV | 12237.6 | 23415.5 | 2214.2 | 2006.2 | 20096.0 | 21298.0 | 762.8 | 1233.1 |
| *Calanus glacialis* | CIII | 218.9 | 341.0 | 93.3 | 85.9 | 10458.8 | 22867.9 | 26.3 | 51.9 |
| *Calanus glacialis* | CII | 5.6 | 13.4 | 335.0 | 366.3 | 5.7 | 15.1 | 0.0 | 0.0 |
| *Calanus glacialis* | CI | 0.0 | 0.0 | 769.0 | 703.3 | 0.0 | 0.0 | 0.0 | 0.0 |
| *Calanus hyperboreus* | AM | 37.8 | 63.3 | 0.0 | 0.0 | 0.0 | 0.0 | 19.2 | 0.0 |
| *Calanus hyperboreus* | AF | 23.6 | 44.9 | 1.0 | 2.0 | 24.6 | 39.7 | 26.0 | 85.9 |
| *Calanus hyperboreus* | CV | 20.4 | 27.4 | 5.0 | 6.0 | 63.4 | 72.7 | 26.4 | 28.2 |
| *Calanus hyperboreus* | CIV | 185.8 | 199.9 | 1.0 | 2.0 | 655.4 | 861.2 | 25.2 | 44.2 |
| *Calanus hyperboreus* | CIII | 44.7 | 134.2 | 1.0 | 2.0 | 60.6 | 103.7 | 5.6 | 25.0 |
| *Calanus hyperboreus* | CII | 2.4 | 7.1 | 46.0 | 76.3 | 0.0 | 0.0 | 0.0 | 0.0 |
| *Calanus hyperboreus* | CI | 14.5 | 35.5 | 0.0 | 0.0 | 0.0 | 0.0 | 2653.3 | 0.0 |
| *Chiridius obtusifrons* |  | 4.9 | 11.8 | 0.0 | 0.0 | 4.0 | 10.6 | 14.4 | 42.5 |
| *Gaetanus brevispinus* |  | 4.4 | 7.9 | 0.0 | 0.0 | 0.6 | 1.5 | 14.8 | 19.7 |
| *Gaetanus tenuispinus* |  | 24.0 | 42.3 | 0.0 | 0.0 | 4.0 | 10.6 | 10.4 | 35.9 |
| *Heterorhabdus norvegicus* |  | 30.7 | 30.6 | 0.0 | 0.0 | 7.4 | 18.0 | 15.2 | 55.9 |
| *Metridia lucens* |  | 24.4 | 43.3 | 5.0 | 7.6 | 9.1 | 24.2 | 123.1 | 13.0 |
| *Metridia longa* | AM | 577.6 | 1230.7 | 212.0 | 227.3 | 209.1 | 356.9 | 315.5 | 160.8 |
| *Metridia longa* | AF | 427.6 | 266.7 | 725.0 | 696.7 | 689.6 | 1322.0 | 365.3 | 322.0 |
| *Metridia longa* | CV | 288.4 | 142.9 | 136.0 | 177.0 | 1251.0 | 1607.2 | 331.2 | 250.6 |
| *Metridia longa* | CIV | 245.8 | 275.0 | 79.5 | 138.5 | 681.3 | 734.7 | 284.4 | 512.4 |
| *Metridia longa* | CIII | 183.3 | 349.3 | 6.0 | 12.0 | 343.1 | 443.7 | 134.8 | 458.9 |
| *Metridia longa* | CII | 28.7 | 59.1 | 4.0 | 8.0 | 141.4 | 133.4 | 26.1 | 41.6 |
| *Metridia longa* | CI | 6.7 | 11.0 | 1.0 | 2.0 | 60.6 | 158.5 | 13.1 | 15.9 |
| *Microcalanus* spp. |  | 16873.4 | 27294.1 | 28377.7 | 37357.1 | 24594.8 | 17408.2 | 6396.0 | 2623.7 |
| *Microsetella norvegica* |  | 9.8 | 14.7 | 0.0 | 0.0 | 68.6 | 181.4 | 1.2 | 3.6 |
| *Paraeuchaeta barbata* |  | 2.2 | 6.7 | 0.0 | 0.0 | 0.6 | 1.5 | 14.4 | 0.0 |
| *Paraeuchaeta* spp. |  | 42.7 | 40.9 | 44.0 | 68.4 | 59.0 | 94.7 | 61.3 | 67.7 |
| *Pleuromamma* |  | 1.8 | 2.1 | 0.0 | 0.0 | 0.0 | 0.0 | 2.4 | 1.8 |
| *Pseudocalanus* spp. |  | 11435.1 | 16734.9 | 1847.5 | 798.5 | 58454.1 | 52809.8 | 3748.4 | 5969.4 |
| *Scaphocalanus magnus* |  | 1.3 | 4.0 | 0.0 | 0.0 | 0.6 | 1.5 | 7.9 | 8.9 |
| *Scolecithricella minor* |  | 14.7 | 20.2 | 14.0 | 28.0 | 0.0 | 0.0 | 51.1 | 21.9 |
| *Spinocalanus* spp. |  | 0.0 | 0.0 | 0.0 | 0.0 | 0.0 | 0.0 | 4.4 | 8.9 |
| *Mormonilla minor* |  | 0.0 | 0.0 | 0.0 | 0.0 | 0.0 | 0.0 | 109.7 | 10.7 |
| *Oithona atlantica* |  | 99.3 | 201.9 | 29.0 | 33.8 | 1144.9 | 1760.8 | 3047.2 | 314.3 |
| *Oithona similis* |  | 20268.6 | 18530.2 | 12254.1 | 15776.1 | 143999.7 | 127660.5 | 3004.3 | 4249.3 |
| *Oncaea* spp. |  | 0.0 | 0.0 | 0.0 | 0.0 | 28.6 | 75.6 | 622.0 | 288.0 |
| *Triconia borealis* |  | 834.5 | 1593.5 | 209.5 | 389.9 | 19622.4 | 21307.8 | 561.6 | 1412.4 |
| Calanoida indet |  | 24.4 | 73.3 | 0.0 | 0.0 | 0.0 | 0.0 | 26.1 | 17.9 |
| Cyclopoida indet. |  | 0.0 | 0.0 | 0.0 | 0.0 | 0.0 | 0.0 | 52.5 | 35.8 |
| Harpacticoida indet. |  | 24.9 | 44.4 | 10.7 | 13.1 | 502.9 | 716.9 | 58.0 | 94.7 |
| Copepoda | nauplii | 566.7 | 913.1 | 36284.7 | 33184.6 | 2647.7 | 2069.7 | 34.9 | 41.9 |
| Ostracoda |  | 628.2 | 1004.3 | 9.0 | 6.8 | 204.4 | 458.6 | 136.8 | 393.7 |
| Amphipod indet, |  | 0.0 | 0.0 | 0.0 | 0.0 | 2.3 | 3.1 | 0.8 | 1.8 |
| *Apherusa glacialis* |  | 0.9 | 1.8 | 0.0 | 0.0 | 10.3 | 27.2 | 1.2 | 3.6 |
| *Themisto abyssorum* |  | 5.8 | 8.0 | 37.0 | 34.6 | 16.0 | 20.8 | 4.8 | 0.0 |
| *Themisto compressa* |  | 0.0 | 0.0 | 0.0 | 0.0 | 0.6 | 1.5 | 21.2 | 13.1 |
| *Themisto libellula* |  | 1.8 | 4.1 | 2.0 | 4.0 | 58.6 | 65.0 | 13.2 | 21.5 |
| *Thysanoessa inermis* |  | 31.1 | 47.4 | 6.0 | 5.2 | 16.6 | 20.6 | 13.6 | 37.8 |
| *Thysanoessa longicaudata* |  | 45.3 | 61.9 | 1.0 | 2.0 | 16.6 | 40.4 | 0.8 | 2.2 |
| *Thysanoessa raschii* |  | 1.3 | 2.8 | 2.0 | 4.0 | 2.1 | 5.7 | 0.0 | 0.0 |
| *Meganyctiphanes norvegica* | | 1.8 | 3.5 | 0.0 | 0.0 | 2.3 | 6.0 | 0.8 | 0.0 |
| *Euphausidacea* | nauplii | 0.0 | 0.0 | 5235.9 | 7586.6 | 0.0 | 0.0 | 45.1 | 13.9 |
| *Euphausidacea* | furcilia | 0.0 | 0.0 | 23.0 | 24.1 | 26.9 | 65.9 | 0.0 | 0.0 |
| *Hymenodora glacialis* |  | 0.4 | 1.3 | 0.0 | 0.0 | 0.0 | 0.0 | 0.4 | 1.8 |
| *Pandalus borealis* | juvenile | 0.0 | 0.0 | 1.0 | 2.0 | 0.6 | 1.5 | 8.1 | 1.8 |
| Isopoda indet. |  | 0.0 | 0.0 | 28.3 | 40.2 | 68.1 | 106.6 | 7.7 | 17.8 |
| *Cirripedia* | nauplii | 0.0 | 0.0 | 112739.6 | 188391.5 | 255.7 | 420.7 | 38.5 | 111.8 |
| Mysidacea indet. |  | 0.9 | 1.8 | 0.0 | 0.0 | 0.0 | 0.0 | 14.4 | 1.8 |
| Decapoda indet. | zoea larvae | 0.0 | 0.0 | 131.0 | 176.6 | 0.0 | 0.0 | 0.0 | 0.0 |
| *Pagurus sp.* | megalopa | 0.0 | 0.0 | 0.0 | 0.0 | 0.6 | 1.5 | 0.0 | 0.0 |
| *Hyas* sp. | megalopa | 0.0 | 0.0 | 0.0 | 0.0 | 0.6 | 1.5 | 0.0 | 0.0 |
| *Aglantha digitale* |  | 6.7 | 6.3 | 1.0 | 2.0 | 115.9 | 141.1 | 14.0 | 35.4 |
| *Sarsia* sp. |  | 0.4 | 1.3 | 0.0 | 0.0 | 0.0 | 0.0 | 0.8 | 0.0 |
| *Hydrozoa* indet. |  | 1.8 | 2.9 | 30.0 | 36.1 | 2.3 | 3.1 | 7.7 | 2.2 |
| *Dimophyes arctica* |  | 13.8 | 21.5 | 4.0 | 5.7 | 4.0 | 6.9 | 9.6 | 40.7 |
| Siphonophora indet. |  | 0.0 | 0.0 | 5.0 | 7.6 | 0.0 | 0.0 | 0.0 | 0.0 |
| Scyphozoa indet. |  | 0.0 | 0.0 | 0.0 | 0.0 | 0.0 | 0.0 | 1.2 | 0.0 |
| *Beröe cucumis* |  | 1.3 | 4.0 | 9.0 | 18.0 | 14.9 | 21.4 | 1.2 | 3.6 |
| *Mertensia ovum* |  | 7.6 | 17.0 | 3.0 | 3.8 | 7.4 | 10.9 | 5.6 | 0.0 |
| Ctenophora indet. | juvenile | 15.1 | 27.0 | 3.0 | 6.0 | 0.0 | 0.0 | 241.5 | 13.1 |
| *Clione limacina* | larvae | 8.0 | 17.0 | 0.0 | 0.0 | 230.3 | 328.6 | 241.1 | 632.8 |
| *Clione limacina* | adult | 0.9 | 1.8 | 0.0 | 0.0 | 56.0 | 144.7 | 25.2 | 16.6 |
| *Limacina helicina* |  | 90.7 | 118.0 | 36.0 | 27.9 | 194137.0 | 289073.3 | 20.0 | 32.9 |
| *Limacina retroversa* |  | 721.8 | 1909.9 | 4.0 | 5.7 | 408.9 | 819.1 | 5.3 | 0.0 |
| *Eukrohnia hamata* |  | 271.1 | 324.5 | 133.3 | 191.3 | 953.1 | 1916.1 | 218.0 | 529.0 |
| *Parasagitta elegans* |  | 184.4 | 168.8 | 150.7 | 153.0 | 560.0 | 419.2 | 48.0 | 94.5 |
| *Parasagitta maxima* |  | 0.0 | 0.0 | 0.0 | 0.0 | 0.6 | 1.5 | 0.0 | 0.0 |
| *Chaetognatha* indet. | juvenile | 0.0 | 0.0 | 12.0 | 14.2 | 0.0 | 0.0 | 4.8 | 0.0 |
| *Fritillaria borealis* |  | 12.4 | 31.5 | 272.7 | 499.6 | 7397.2 | 11470.6 | 18.1 | 21.5 |
| *Oikopleura* spp. |  | 47.6 | 83.7 | 6.0 | 5.2 | 377.7 | 311.9 | 13.3 | 25.1 |
| Polychaeta indet. |  | 0.0 | 0.0 | 0.0 | 0.0 | 13.7 | 36.3 | 6.0 | 5.4 |
| Polychaeta | larvae | 18.7 | 32.6 | 9390.0 | 15876.8 | 171.1 | 78.4 | 6.5 | 10.4 |
| Echinodermata | larvae | 18.7 | 37.0 | 6.7 | 13.3 | 1966.6 | 3378.1 | 147.4 | 26.8 |
| Bryozoa | larvae | 2.7 | 5.3 | 0.0 | 0.0 | 32.9 | 45.7 | 23.2 | 37.6 |
| Anthozoa | larvae | 0.0 | 0.0 | 24.0 | 38.1 | 14.3 | 37.8 | 2.0 | 1.8 |
| Bivalvia | juvenile | 0.0 | 0.0 | 0.0 | 0.0 | 26113.1 | 39153.1 | 3.2 | 7.2 |
| DEAD |  |  |  |  |  |  |  |  |  |
| *Acartia longiremis* |  | 0.4 | 1.3 | 0.0 | 0.0 | 85.7 | 200.9 | 14.4 | 17.5 |
| *Bradydius similis* |  | 1.8 | 5.3 | 12.0 | 24.0 | 22.9 | 60.5 | 44.3 | 10.4 |
| *C. hyperboreus* | CIII | 0.0 | 0.0 | 0.0 | 0.0 | 0.0 | 0.0 | 7.6 | 30.4 |
| *C. hyperboreus* | CIV | 0.9 | 2.7 | 0.0 | 0.0 | 52.6 | 106.2 | 5.2 | 11.8 |
| *C. hyperboreus* | CV | 0.0 | 0.0 | 0.0 | 0.0 | 0.0 | 0.0 | 1.6 | 3.6 |
| *C. hyperboreus* | AF | 2.2 | 5.3 | 0.0 | 0.0 | 0.0 | 0.0 | 8.4 | 7.2 |
| *Calanus* spp. | CI | 0.0 | 0.0 | 27.3 | 49.5 | 0.0 | 0.0 | 0.0 | 0.0 |
| *Calanus* spp. | CII | 0.0 | 0.0 | 12.3 | 15.4 | 0.0 | 0.0 | 4.4 | 0.0 |
| *Calanus* spp. | CIII | 16.9 | 22.2 | 9.0 | 18.0 | 593.1 | 1227.5 | 419.3 | 14.0 |
| *Calanus* spp. | CIV | 2398.8 | 2022.6 | 22.0 | 36.4 | 1330.4 | 1042.7 | 1043.5 | 980.9 |
| *Calanus* spp. | CV | 3472.0 | 3251.6 | 33.0 | 45.8 | 633.7 | 583.8 | 648.1 | 630.9 |
| *Calanus* spp. | AF | 111.6 | 130.1 | 188.0 | 322.1 | 40.6 | 68.1 | 36.8 | 13.7 |
| *Calanus* spp. | AM | 78.0 | 90.0 | 9.0 | 15.4 | 0.0 | 0.0 | 34.4 | 23.3 |
| *Chiridius obtusifrons* |  | 0.0 | 0.0 | 0.0 | 0.0 | 0.0 | 0.0 | 0.8 | 1.8 |
| *Gaetanus* spp. |  | 0.0 | 0.0 | 0.0 | 0.0 | 0.0 | 0.0 | 0.8 | 1.8 |
| *Heterorhabdus norvegicus* |  | 0.0 | 0.0 | 0.0 | 0.0 | 0.0 | 0.0 | 1.6 | 1.8 |
| *Metridia longa* |  | 173.1 | 165.9 | 22.5 | 18.9 | 70.4 | 87.8 | 393.3 | 482.5 |
| *Microcalanus* spp. |  | 5920.3 | 5748.9 | 1461.4 | 1134.1 | 2097.6 | 1322.9 | 1167.5 | 888.7 |
| *Pareuchaeta* spp. |  | 4.9 | 10.3 | 1.0 | 2.0 | 4.0 | 8.9 | 8.1 | 3.6 |
| *Pseudocalanus* spp. |  | 3005.8 | 3211.7 | 45.0 | 28.7 | 2402.4 | 1870.6 | 1755.9 | 323.0 |
| *Scaphocalanus magnus* |  | 0.0 | 0.0 | 0.0 | 0.0 | 0.0 | 0.0 | 0.8 | 1.8 |
| *Scolecitricella minor* |  | 0.0 | 0.0 | 0.0 | 0.0 | 0.0 | 0.0 | 2.4 | 5.4 |
| *Spinocalanus* spp. |  | 0.0 | 0.0 | 0.0 | 0.0 | 0.0 | 0.0 | 6.8 | 28.6 |
| *Oithona atlantica* |  | 0.0 | 0.0 | 0.0 | 0.0 | 47.4 | 72.3 | 45.9 | 80.6 |
| *Oithonas similis* |  | 2792.9 | 2231.2 | 2217.9 | 2238.2 | 18123.1 | 17717.4 | 1757.2 | 1982.2 |
| *Triconia borealis* |  | 16.9 | 34.3 | 0.0 | 0.0 | 396.6 | 371.4 | 197.1 | 494.1 |
| Copepoda | nauplii | 0.0 | 0.0 | 60.0 | 104.6 | 6.3 | 14.9 | 13.9 | 19.0 |
| Euphausidacea indet. |  | 11.1 | 15.1 | 0.0 | 0.0 | 0.6 | 1.5 | 6.8 | 12.2 |
| Euphausidacea | nauplii | 0.0 | 0.0 | 70.0 | 140.0 | 0.0 | 0.0 | 62.1 | 0.0 |
| Chaetognatha indet. |  | 46.7 | 59.4 | 13.0 | 23.4 | 164.0 | 292.8 | 124.3 | 130.1 |

**Supplementary Table SIII**. Model selection in search of the most parsimonious model explaining the observed carcass abundance, biomass and percent dead of *Calanus* spp, Other Copepods and carcass abundance and percent dead of *Pseudocalanus* spp., *Oithona similis* and *Microcalanus* spp. (**bolded**). Predictor variables included: sampling month (SM, four-level factor: January 2016, May 2016, August 2016 and January 2017) and water mass (WM, 5-level factor: (Arctic Water (T<1^o^C, S<34.65), Intermediate water (T>1^o^C, S 34.0-34.65), Transformed Atlantic Water (T=1-3^o^C, S>34.65) or Atlantic Water (T>3^o^C, S>34.65)). The most parsimonious model is listed at the top and presented the lowest AIC_c_-value, Δi is the differences in AIC score between the best model and the model being compared, w_i_ refers to the weight of the model, and K represents the number of parameters in the model.

| Model names | K | AICc | Δi | wi | Model names | K | AICc | Δi | wi |
| --- | --- | --- | --- | --- | --- | --- | --- | --- | --- |
| Abundance | | | | | **Percent Dead** | | | | |
| *Calanus* spp. |  |  |  |  |  |  |  |  |  |
| SM | 5 | 453.2 | 0.0 | 0.98 | SM | 5 | -64.1 | 0.0 | 0.99 |
| SM+WM | 8 | 460.7 | 7.5 | 0.02 | SM+WM | 8 | -54.4 | 9.7 | 0.01 |
| WM | 5 | 466.3 | 13.1 | 0.00 | WM | 5 | -45.8 | 18.3 | 0.00 |
| Other Copepods | |  |  |  |  |  |  |  |  |
| SM | 5 | 515.6 | 0.0 | 0.95 | SM | 5 | -54.3 | 0.0 | 0.99 |
| SM+WM | 8 | 522.4 | 6.8 | 0.03 | SM+WM | 8 | -44.0 | 10.3 | 0.01 |
| WM | 5 | 524.2 | 8.6 | 0.01 | WM | 5 | -40.4 | 13.9 | 0.00 |
| *Pseudocalanus* spp*.* | |  |  |  |  |  |  |  |  |
| SM | 5 | 416.6 | 0.0 | 0.98 | SM | 5 | 416.6 | 0.0 | 0.98 |
| SM+WM | 8 | 424.3 | 7.6 | 0.02 | SM+WM | 8 | 424.3 | 7.6 | 0.02 |
| WM | 5 | 441.9 | 25.3 | 0.00 | WM | 5 | 441.9 | 25.3 | 0.00 |
| Oithona similis | |  |  |  |  |  |  |  |  |
| SM | 5 | 479.7 | 0.0 | 0.99 | SM | 5 | -57.3 | 0.0 | 0.96 |
| SM+WM | 8 | 488.9 | 9.3 | 0.01 | WM | 5 | -50.0 | 7.3 | 0.02 |
| WM | 5 | 491.2 | 11.5 | 0.00 | SM+WM | 8 | -49.0 | 8.3 | 0.02 |
| *Microcalanus* spp*.* | |  |  |  |  |  |  |  |  |
| SM | 5 | 456.6 | 0.0 | 0.95 | SM | 5 | -32.2 | 0.0 | 0.92 |
| WM | 5 | 462.7 | 6.1 | 0.05 | SM+WM | 8 | -27.2 | 5.0 | 0.08 |
| SM+WM | 8 | 467.5 | 10.9 | 0.00 | WM | 5 | -16.7 | 15.4 | 0.00 |
| *Metridia longa* | |  |  |  |  |  |  |  |  |
| SM | 5 | 296.6 | 0.0 | 0.96 | WM | 5 | -50.7 | 0.0 | 0.75 |
| WM | 5 | 303.3 | 6.8 | 0.03 | SM | 5 | -48.4 | 2.3 | 0.24 |
| SM+WM | 8 | 305.6 | 9.0 | 0.01 | SM+WM | 8 | -40.0 | 10.7 | 0.00 |
| *Triconia borealis* | |  |  |  |  |  |  |  |  |
| SM | 5 | 215.7 | 0.0 | 0.94 | SM | 5 | -113.4 | 0.0 | 0.97 |
| SM+WM | 8 | 221.2 | 5.5 | 0.06 | SM+WM | 8 | -106.6 | 6.8 | 0.03 |
| WM | 5 | 310.0 | 94.2 | 0.00 | WM | 5 | -90.4 | 23.0 | 0.00 |
| Biomass | | | | |  |  |  |  |  |
| *Calanus* spp. | |  |  |  |  |  |  |  |  |
| SM | 5 | 453.2 | 0.0 | 0.98 | SM | 5 | -53.3 | 0.0 | 0.97 |
| SM+WM | 8 | 460.7 | 7.5 | 0.02 | WM | 8 | -45.7 | 7.5 | 0.02 |
| WM | 5 | 466.3 | 13.1 | 0.00 | SM+WM | 5 | -41.9 | 11.3 | 0.00 |
| Other Copepods | | |  |  |  |  |  |  |  |
| SM | 5 | 276.1 | 0.0 | 0.99 | SM | 5 | -54.9 | 0.0 | 0.95 |
| SM+WM | 8 | 287.5 | 11.3 | 0.00 | WM | 5 | -48.5 | 6.3 | 0.04 |
| WM | 5 | 288.9 | 12.8 | 0.00 | SM+WM | 8 | -46.0 | 8.8 | 0.01 |

**Supplementary Table SIV**. Model selection in search of the most parsimonious model explaining the observed carcass abundance and percent dead of *Calanus* spp. and Other Copepods in different depth layers. Predictor variables included: sampling month (SM, four-level factor: January 2016, May 2016, August 2016 and January 2017) and water mass (WM, 5-level factor: (Arctic Water (T<1^o^C, S<34.65), Intermediate water (T>1^o^C, S 34.0-34.65), Transformed Atlantic Water ( T=1-3^o^C, S>34.65) or Atlantic Water (T>3^o^C, S>34.65)), and sampling layer (SL, 5-level factor (0-20 m, 20-50 m, 50-100 m, 100 -200m/bottom, 200m-bottom). The most parsimonious model is listed at the top and presented the lowest AIC_c_-value, Δi is the differences in AIC score between the best model and the model being compared, w_i_ refers to the weight of the model, and K represents the number of parameters in the model.

| Model names | K | AICc | Δi | wi | Model names | K | AICc | Δi | wi |
| --- | --- | --- | --- | --- | --- | --- | --- | --- | --- |
| Abundance | | | | | Proportion | | | | |
| *Calanus* |  |  |  |  |  |  |  |  |  |
| SM | 5 | 728.1 | 0.0 | 0.53 | SM | 5 | -246.7 | 0.0 | 0.75 |
| SM+SL | 9 | 730.4 | 2.3 | 0.17 | SM+SL | 9 | -243.5 | 3.1 | 0.16 |
| SM+WM | 9 | 730.6 | 2.5 | 0.15 | SM+WM | 9 | -242.0 | 4.7 | 0.07 |
| SM+WM+SL | 13 | 730.7 | 2.6 | 0.15 | SM+WM+SL | 14 | -239.9 | 6.8 | 0.02 |
| WM | 6 | 770.2 | 42.1 | 0.00 | SL | 6 | -226.4 | 20.3 | 0.00 |
| WM+SL | 10 | 774.5 | 46.4 | 0.00 | WM | 6 | -225.6 | 21.0 | 0.00 |
| SL | 6 | 776.1 | 48.0 | 0.00 | WM+SL | 10 | -221.0 | 25.7 | 0.00 |
| Other cop |  |  |  |  |  |  |  |  |  |
| SM+SL | 9 | 1143.0 | 0.0 | 0.57 | SM | 5 | -195.4 | 0.2 | 0.35 |
| SM+WM+SL | 13 | 1143.6 | 0.6 | 0.42 | SM+WM | 9 | -195.5 | 0.0 | 0.39 |
| WM+SL | 10 | 1152.0 | 9.0 | 0.01 | SM+WM+SL | 14 | -193.6 | 1.9 | 0.15 |
| SM | 5 | 1153.4 | 10.4 | 0.00 | SM+SL | 10 | -193.1 | 2.4 | 0.11 |
| SM+WM | 9 | 1154.9 | 11.9 | 0.00 | WM | 6 | -168.2 | 27.3 | 0.00 |
| WM | 6 | 1155.0 | 12.0 | 0.00 | SL | 7 | -162.9 | 32.7 | 0.00 |
| SL | 6 | 1184.4 | 41.4 | 0.00 | WM+SL | 11 | -159.4 | 36.1 | 0.00 |

**Supplementary Table SV:** Parameter estimates for the generalized linear model (GLM) that best predicts the depth resolved carcass abundance and percent dead of *Calanus* spp. and Other Copepods. For model selection see Supplementary Table SIV. Predictor variables include sampling month (January 2016, May 2016, August 2016 and January 2016) with January 2016 set as reference, and water mass (WM, ArW=Arctic Water (T<1^o^C, S<34.65), IW= Intermediate water (T>1^o^C, S 34.0-34.65), TAW=Transformed Atlantic Water ( T=1-3^o^C, S>34.65), AW= Atlantic Water (T>3^o^C, S>34.65)), with ArW set as reference. Confidence intervals are given in squared brackets. GLM of abundance was run with a gamma distribution, percent dead with beta regression. *** p < 0.001; ** p < 0.01; * p < 0.05

|  | Abundance | | Precent Dead | |
| --- | --- | --- | --- | --- |
|  | ***Calanus* spp.** | **Other Copepods** | ***Calanus* spp.** | **Other Copepods** |
| Intercept | 26.98 *** | 54.87 *** | -0.94 *** | -1.12 *** |
|  | [16.94, 42.97] | [35.64, 84.47] | [-1.27, -0.61] | [-1.35, -0.89] |
| May16 | 0.05 *** | 0.37 ** | -1.03 *** | -0.87 *** |
|  | [0.02, 0.12] | [0.19, 0.74] | [-1.60, -0.45] | [-1.32, -0.42] |
| Aug16 | 0.27 *** | 1.48 | -1.11 *** | -0.97 *** |
|  | [0.13, 0.54] | [0.86, 2.55] | [-1.60, -0.62] | [-1.35, -0.59] |
| Jan17 | 0.21 *** | 0.49 * | -0.17 | -0.01 |
|  | [0.10, 0.45] | [0.29, 0.85] | [-0.68, 0.34] | [-0.37, 0.35] |
| WM- AW |  | 0.56 * |  |  |
|  |  | [0.34, 0.93] |  |  |
| WM -IW |  | 0.97 |  |  |
|  |  | [0.45, 2.12] |  |  |
| WM -SW |  | 2.69 * |  |  |
|  |  | [1.12, 6.47] |  |  |
| WM -TAW |  | 0.60 |  |  |
|  |  | [0.31, 1.16] |  |  |
| (phi) |  |  | 2.46 *** | 7.63 *** |
|  |  |  | [1.83, 3.09] | [5.71, 9.54] |
| N | 122 | 122 | 122 | 122 |
| AIC | 727.55 | 1141.40 | -247.20 | -195.90 |
| pseudo R^2^ | 0.33 | 0.39 | 0.23 | 0.20 |
| df.null |  |  | 120.00 | 120.00 |
| logLik |  |  | 128.60 | 102.95 |
| df.residual |  |  | 117.00 | 117.00 |

**Supplementary Table SVI:** Mean lipid content (µg), Wax ester (WE) carbon content (µg) and lipid sac area (LA) to prosome area (PA) ratio of live CIV, CV and adult females (AF) of *C. glacialis* and *C. finmarchicus* in January, May and August 2016 and January 2017. Lipid and wax ester content were estimated from LA measurements of digital images of individual *Calanus* following Vogedes *et al.* (2010). Wax ester was converted to carbon using a factor of 0.8 (Kattner and Hagen, 2009). SD: standard deviation. For lipid content, the first quartile and maximum values are also shown. N is the number of individuals that were analysed.

|  |  |  | Lipid content (µg) | | | | Carbon WE (µg) | | LA/PA ratio | |
| --- | --- | --- | --- | --- | --- | --- | --- | --- | --- | --- |
| Stage | **Date** | **N** | Mean | SD | 1st quartile | Max. | Mean | SD | Mean | SD |
| *C. glacialis* | |  |  |  |  |  |  |  |  |  |
| AF | Jan-16 | 172 | 222 | 97 | 166 | 652 | 151 | 68 | 0.37 | 0.09 |
|  | May | 9 | 98 | 102 | 2 | 284 | 66 | 70 | 0.21 | 0.18 |
|  | August | 335 | 360 | 133 | 283 | 741 | 249 | 94 | 0.50 | 0.12 |
|  | Jan-17 | 87 | 253 | 98 | 185 | 556 | 173 | 69 | 0.39 | 0.07 |
| CV | Jan-16 | 196 | 102 | 57 | 57 | 269 | 68 | 39 | 0.29 | 0.10 |
|  | May | 7 | 136 | 89 | 76 | 255 | 92 | 62 | 0.32 | 0.12 |
|  | August | 393 | 249 | 167 | 95 | 692 | 172 | 117 | 0.45 | 0.17 |
|  | Jan-17 | 278 | 139 | 62 | 93 | 431 | 93 | 43 | 0.37 | 0.10 |
| CIV | Jan-16 | 389 | 33 | 20 | 19 | 157 | 21 | 13 | 0.22 | 0.09 |
|  | May | 62 | 39 | 23 | 21 | 106 | 25 | 15 | 0.25 | 0.09 |
|  | August | 268 | 31 | 29 | 6 | 141 | 20 | 19 | 0.21 | 0.14 |
|  | Jan-17 | 9 | 43 | 29 | 26 | 107 | 28 | 20 | 0.26 | 0.10 |
| *C. finmarchicus* | |  |  |  |  |  |  |  |  |  |
| AF | Jan-16 | 31 | 148 | 71 | 93 | 310 | 100 | 49 | 0.37 | 0.11 |
|  | May | 14 | 18 | 21 | 4 | 65 | 12 | 14 | 0.09 | 0.07 |
|  | August | 35 | 140 | 116 | 28 | 478 | 95 | 80 | 0.33 | 0.21 |
|  | Jan-17 | 16 | 189 | 99 | 139 | 414 | 128 | 68 | 0.37 | 0.12 |
| CV | Jan-16 | 394 | 96 | 64 | 53 | 369 | 64 | 44 | 0.39 | 0.12 |
|  | May | 3 | 28 | 13 | 24 | 37 | 18 | 8 | 0.16 | 0.03 |
|  | August | 61 | 123 | 113 | 10 | 504 | 84 | 78 | 0.36 | 0.23 |
|  | Jan-17 | 559 | 136 | 87 | 62 | 458 | 92 | 60 | 0.43 | 0.14 |
| CIV | Jan-16 | 138 | 34 | 19 | 20 | 98 | 22 | 13 | 0.30 | 0.11 |
|  | May | 4 | 27 | 25 | 11 | 63 | 18 | 16 | 0.24 | 0.12 |
|  | August | 64 | 16 | 22 | 0 | 91 | 10 | 15 | 0.15 | 0.16 |
|  | Jan-17 | 8 | 49 | 24 | 34 | 82 | 32 | 16 | 0.30 | 0.08 |


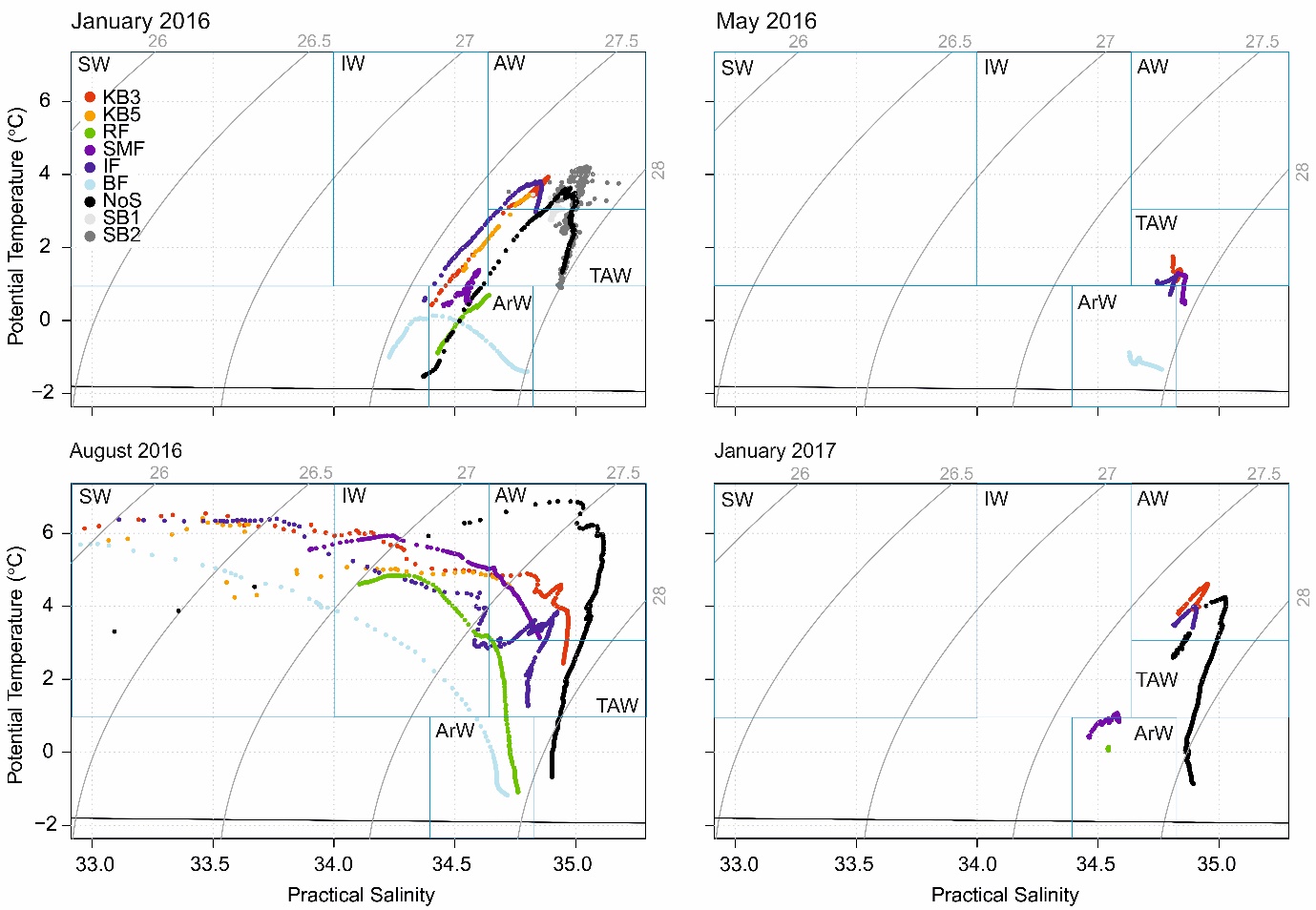


**Supplementary** **Figure S1**: TS-diagram of water masses at all stations sampled in January 2016, May 2016, August 2016 and January 2017. Water masses as defined by Cottier *et al*. (2005) are indicated by light blue boxes. ArW: Arctic Water (T<1^o^C, S<34.65), IW: Intermediate water (T>1^o^C, S 34.0-34.65), TAW: Transformed Atlantic Water (T=1-3^o^C, S>34.65). AW: Atlantic Water (T>3^o^C, S>34.65). Black line indicates freezing point. Grey lines show isopycnals at 0.5 intervals.


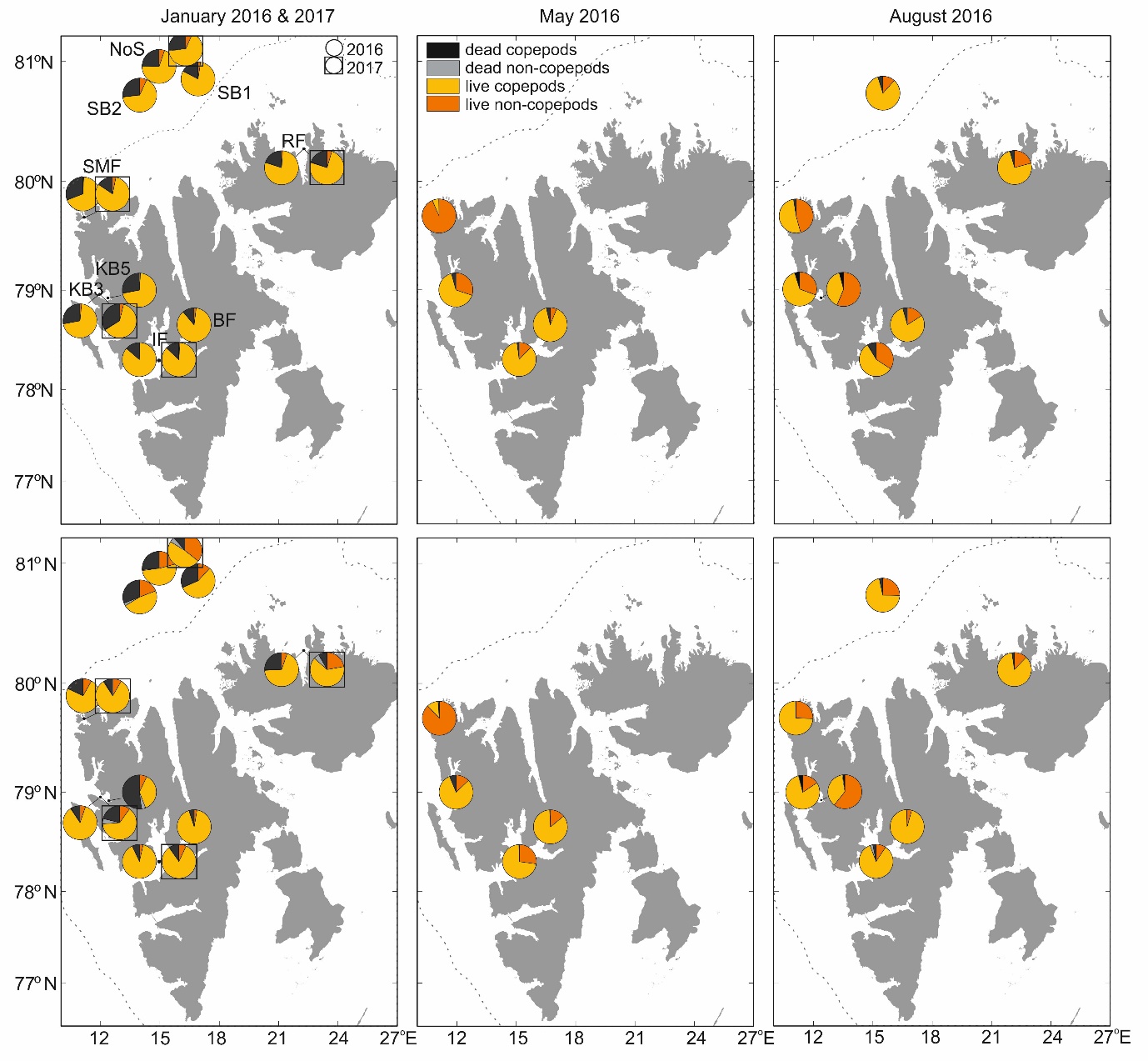


**Supplementary Fig. S2:** Contribution of dead and live copepods and non-copepods at each station in January 2016 and 2017, May 2016 and August 2016 based on abundance (upper panel) and biomass (lower panel). Pies in squared boxes mark samples taken in January 2017. Dotted line marks shelf break (500 m bathymetry line).

BF Billefjorden, IF Isfjorden, KB3/5 Kongsfjorden, SMF Smeerenburgfjorden, SB Shelf break, NOS North of Svalbard, RF Rijpfjorden.


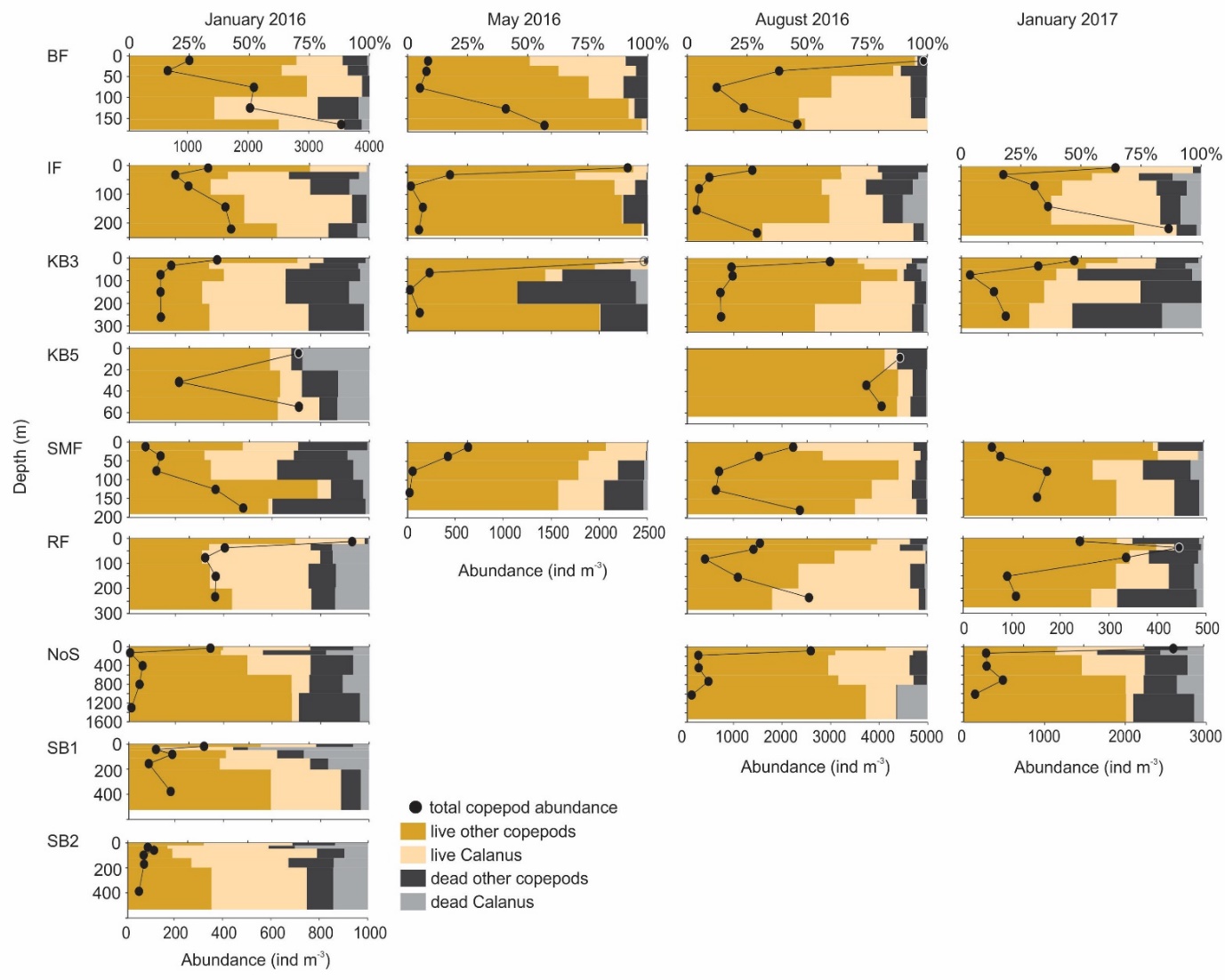


**Supplementary Fig. S3:** Vertical distribution of dead and live copepods at each station. Line and dots show total copepod abundance (live and dead) at each depth (ind. m^-3^). Note differences in scale of abundance (bottom x-axis) between seasons and for BF in January 2016.


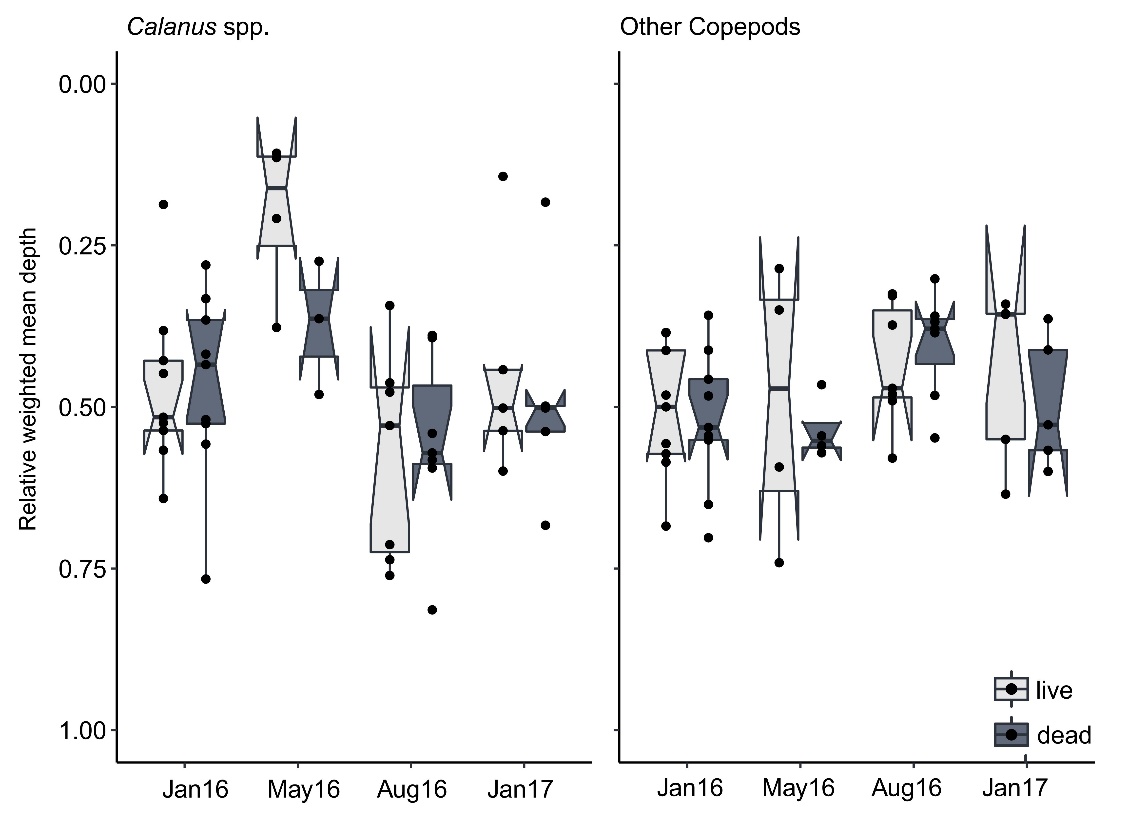


**Supplementary Fig. S4:** Differences in relative weighted mean depth (RZ_m_) of live and dead *Calanus* spp. and Other Copepods in each month. Dots show data points. Horizontal line, whiskers, circles and notches as in Fig. 3.


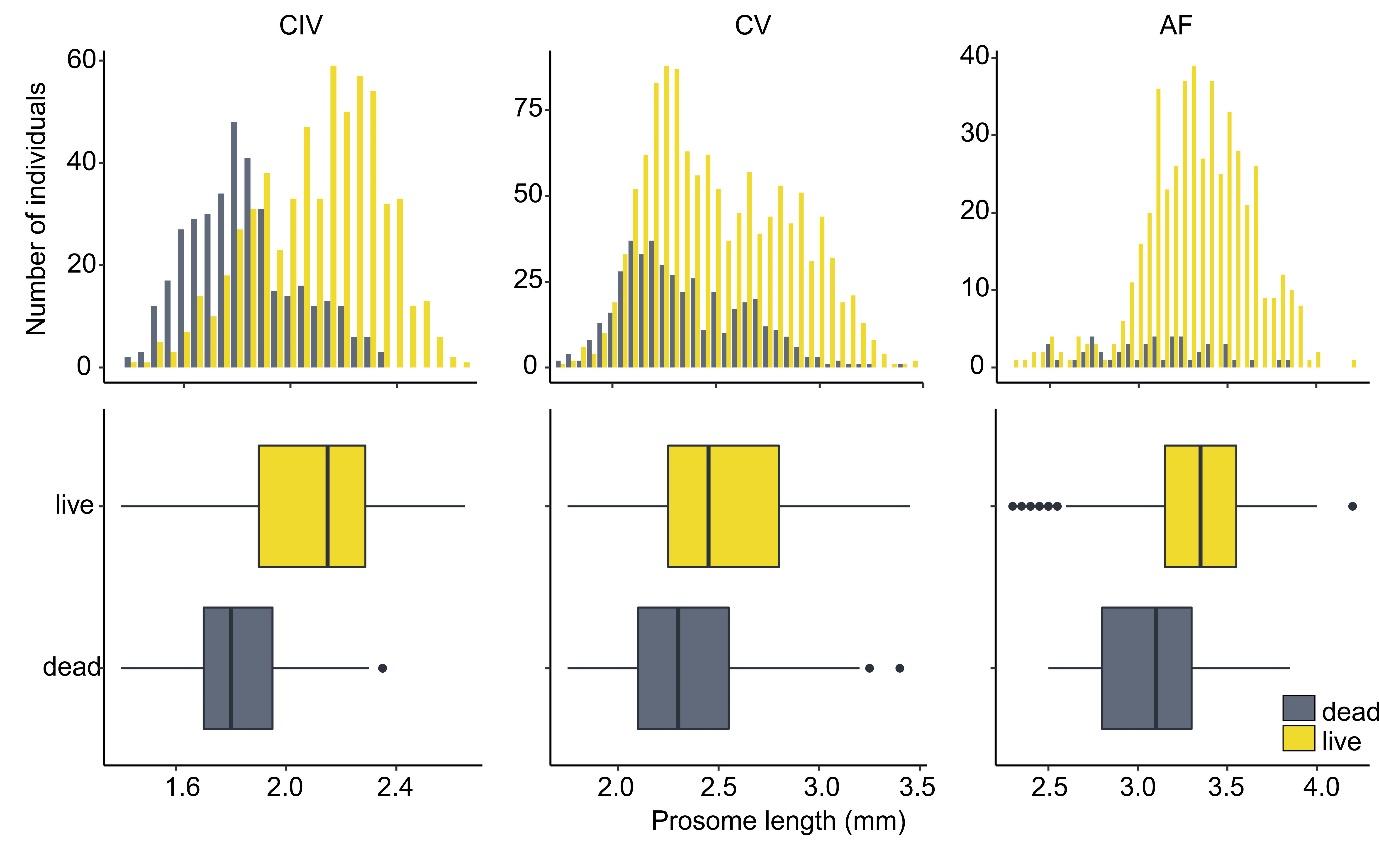


**Supplementary Fig. S5:** Prosome length (mm) frequency distribution of live (yellow) and dead (grey) (upper panel) and boxplot of prosome length (lower panel) of live and dead *Calanus* CIV, CV and adult females (AF) in January 2017. Difference in prosome length were significant between dead and live CIV (Kruskal Wallis, chi sq. 259.91, p<0.001), CV (chi sq. 84.021, p<0.001) and AF (chi sq. 24.089 p<0.001).

**References**

Cottier, F., Tverberg, V., Inall, M., Svendsen, H., Nilsen, F. and Griffiths, C. (2005) Water mass modification in an Arctic fjord through cross-shelf exchange: The seasonal hydrography of Kongsfjorden, Svalbard. *J. Geophys. Res.- Oceans,* **110,** C12005, doi:10.1029/2004JC002757.

Kattner, G. and Hagen, W. (2009) Lipids in marine copepods: latitudinal characteristics and perspective to global warming. In: M. Kainz, M. T. Brett and M. T. Arts (eds) *Lipids in Aquatic Ecosystems.* Springer New York, New York, NY, pp. 257-280.

Vogedes, D., Varpe, Ø., Søreide, J. E., Graeve, M., Berge, J. and Falk-Petersen, S. (2010) Lipid sac area as a proxy for individual lipid content of arctic calanoid copepods. *J. Plankt. Res.,* **32,** 1471-1477.
